# Cholesterol and Alzheimer’s Disease Risk: A Meta-Meta-Analysis

**DOI:** 10.3390/brainsci10060386

**Published:** 2020-06-18

**Authors:** Olalla Sáiz-Vazquez, Alicia Puente-Martínez, Silvia Ubillos-Landa, Joaquín Pacheco-Bonrostro, Javier Santabárbara

**Affiliations:** 1 Department of Occupational Therapy, Faculty of Health Science, University of Burgos, C/Villadiego, 1, 09001 Burgos, Spain; osaiz@ubu.es; 2 Department of Social Psychology and Methodology of Behavioral Science, University of the Basque Country, Avenida Tolosa 70, 20018 San Sebastián, Spain; alicia.puente@ehu.es; 3 Department of Social Psychology, Faculty of Health Science, University of Burgos, C/Villadiego, 1, 09001 Burgos, Spain; 4 Department of Applied Economy, Faculty of Economics and Business Sciences, University of Burgos, Pza. De la Infanta Dª Elena, s/n. 09001 Burgos, Spain; jpacheco@ubu.es; 5 Department of Microbiology, Pediatrics, Radiology and Public Health, University of Zaragoza, C/Domingo Miral s/n, 50009 Zaragoza, Spain; jsantabarbara@unizar.es; 6 Aragonese Institute of Health Sciences (IIS Aragón), 50009 Zaragoza, Spain

**Keywords:** Alzheimer’s disease, etiology, cholesterol, risk factors, meta-analysis

## Abstract

Background: Alzheimer’s disease (AD) is the most common subtype of dementia. In the last ten years, the relationship between cholesterol and AD has been investigated. Evidence suggests that cholesterol is associated with AD and represents promising targets for intervention. However, the causality of these associations is unclear. Therefore, we sought to conduct a meta-meta-analysis to determine the effect of cholesterol on the development AD. Then, we assessed the effect of serum levels of low-density lipoprotein cholesterol (LDL-C) and high-density lipoprotein cholesterol (HDL-C), total cholesterol (TC) and triglycerides (TG), on AD risk. Methods: A systematic search of meta-analyses was conducted. Scopus, Web of Science, Science direct, PubMed and Google academic system databases were reviewed. Results: We found 100 primary studies and five meta-analyses to analyze the relationships between cholesterol and AD. The total effect of cholesterol on risk of AD was significant and heterogeneous. Subgroup analysis shows that LDL-C levels influence the development of AD. However, non-significant effects of HDL-C, TC and TG levels on AD were found. Conclusions: These results strengthen the evidence that LDL-C cholesterol levels increase risk for AD. More initiatives to investigate the relationship between cholesterol and AD are needed.

## 1. Introduction

Alzheimer’s disease (AD) is the most common neurodegenerative disorder resulting in cognitive impairment. AD is characterized by a gradual decline in memory and other cognitive and executive functions, and the progressive development of affective and behavioral disorders [1]. The onset of AD is insidious, and its progression is gradual. As it progresses, various patterns of deficits are seen, but the disorder most commonly begins with deficits in recent memory, which are followed by aphasia, apraxia and agnosia after several years [2]. AD also may cause psychiatric symptoms and personality changes [3]. At the beginning, it affects some abilities, but in the most severe stages, people may depend entirely on others for basic activities of daily living [2]. 

The etiology of AD is unknown [4]. With the global population aging, AD has increased considerably and become a primary concern for governments and the scientific and medical communities [5]. In Europe, the AD rate is around 5.05% (3.31% for men and 7.13% for women). The AD increase by age reaches 4% of prevalence worldwide, and it increases to 4.02% in people over 60 years old [6,7]. A recent study indicated that the prevalence of AD in individuals aged 60 to 69 years was 1.9 times higher in females than in males (108 cases versus 56 cases per 10,000 persons) [7]. In Spain, around 400,000 people suffer from AD, with the highest prevalence in central and north-eastern Spain [8]. 

Disorders of lipid homeostasis are common risk factors for cardiovascular disease, which is linked to AD [9]. Dyslipidemia has been identified as a risk factor for AD [1]. This concept refers to abnormal levels of lipids or lipoproteins in the blood, which include high levels of low-density lipoprotein (LDL-C), low levels of high-density lipoprotein (HDL-C), total cholesterol (TC) and triglycerides (TG) [1]. According to previous results, the overall performance of four independent test results should be considered indexes for the prediction of AD, and provide accurate information on an individual’s lipid metabolism status or serum lipid and cholesterol levels [10,11,12]. 

In the last ten years, the relationship between cholesterol and AD has been extensively investigated, especially in longitudinal epidemiological studies [10]. Evidence suggests that there is a relationship between having high cholesterol levels in blood in mid- and late-life and the development of dementia [1,13]. Specifically, some studies have demonstrated that dyslipidemia, mainly a high level of LDL-C, has vascular and neurotoxic effects, and is implicated in the pathogenesis of AD [10,14,15,16]. Additionally, another study indicates that if the TC in the brain membrane increases, synapses are not performed normally and, therefore, affect cognitive degeneration in AD [17]. Nevertheless, other studies did not find an association between hypercholesterolemia (high levels of LDL-C, TC, and TG) and AD [18,19]. Regarding HDL-C levels, Tynkkynen et al. [20] found that high levels of HDL-C were inversely associated with the risk of AD. Other studies share the same finding [21,22]. However, some studies did not find an association between high triglycerides levels and high levels of HDL-Cproteins and AD [1,12,18].

The study of the disorders of lipid homeostasis is essential, because it may reduce the consequences of vascular diseases and neurodegenerative diseases, among others, in a cost-effective way [1]. First, this study aimed to conduct a meta-meta-analysis to determine the global effect of cholesterol on AD risk. Second, as there was no consensus in the previous literature about the impact of different types of cholesterol on AD, the effects of serum levels of LDL-C, HDL-C, TCTG on the development of AD were analyzed.

## 2. Materials and Methods

### 2.1. Data Collection 

We applied the Preferred Reporting Items for Systematic reviews and Meta-Analyses (PRISMA) guidelines for systematic reviews and meta-analyses [23]. For data collection, we searched meta-analyses reporting outcomes in individuals with diagnoses of AD. To locate potentially suitable studies, we conducted several searches using 5 electronic databases (last search completed in January 2020), including the Web of Science, Scopus, Pubmed, Science Direct and Google Scholar. No publication date was imposed. The electronic search adopted several combinations of the following keywords: “cholesterol” AND Alzheimer’s disease AND meta-analysis. The same search strategy was used in academic Google, but limited to the title. Articles were also searched manually and, if required and when feasible, authors were contacted directly for additional information. The search was also done in the Spanish language. 

The study selection included previous meta-analyses that met the following criteria: (1) meta-analysis studies that included measures for cholesterol (LDL-C, HDL-C, TC and TG) and AD diagnosis; (2) they should be written in English or Spanish; (3) quantitative studies that reported effect sizes or data that enabled effect size calculation or estimation; (4) meta-analyses that included human samples.

All abstracts were independently analyzed by 2 researchers. Then, after the exclusion of irrelevant abstracts, all remaining articles were critically inspected to check data accuracy. For meta-analyses that met the inclusion criteria, a third investigator independently extracted the salient data. Data were collected directly from the text, correlation matrixes or other statistical tables from the included studies (see supplemental material). 

The primary variable (type of cholesterol), design (cross-sectional or longitudinal studies), country of origin of the study, sample size, gender, mean age, main results and an effect size of the relationships between cholesterol and AD were extracted. Information on all the collected data from the selected studies is presented in Table 1.

### 2.2. Quality Assessment

Quality of the meta-analyses was independently coded by two co-authors using the 11-item Assessment of Multiple Systematic Reviews (AMSTAR) tool [24], which has shown to have good inter-rater agreement, reliability, and content validity [24,25]. Total scores for each meta-analysis were calculated as the sum of the 11 items on a binary scale. Quality classifications were established as low quality (0–4), moderate quality (5–8), and high quality (9–11).

### 2.3. Statistical Analysis

We conducted meta-meta-analysis, combining standard mean difference (SMD), odds ratio (OR), and risk ratio (RR) for AD reported in the selected meta-analyses [26]. We report separate meta-analytic results for each meta-analysis in Table 1. Additionally, we identified separate effect sizes for LDL-C, HDL-C, TC and TG cholesterol levels and their relationship with AD risk. The most frequently reported measure of the associations with cholesterol was SMD and OR. Hence, the results of this meta-meta-analysis are reported in OR format. For each meta-analysis, we calculated (see Tables 2–5): (a) the 95% confidence interval of the effect; (b) the *Z*-value and *p* (two-tailed significance); and (c) *k* or number of studies [27]. RRs and ORs were considered as equivalent, as deemed appropriate when the outcome condition is relatively rare (incidence < 15%) [28]. Adjusted effect measures were used in the analysis when they were included in the source studies, under the assumption that adjustment was performed to remove bias in the estimate of the association between cholesterol and AD. We conducted a random-effect model that allowed SMD and ORs to be incorporated into the same input. Random-effect models are more appropriate than fixed-effect models when the number of studies included in the meta-analysis is low (< 10) [29].

Initially, we performed an analysis summarizing all the available data into a single pooled estimate [30]. Then, to assess the heterogeneity of our results, subgroup analyses were performed to examine the differential effects of type of cholesterol: (1) LDL-C, (2) HDL-C, (3) TC and (4) TG. We did not assume a common among-study variance component across subgroups. 

We calculated summary estimates and plotted the effects, using Comprehensive Meta-Analysis software [31]. The heterogeneity of the results obtained from the different meta-analysis was calculated using the *Q* statistic. Additionally, the presence of heterogeneity was evaluated by calculating the *I*^2^. The *I*^2^ statistic explains the percentage of variance in the observed effects due to variance in the true effects. *I*^2^ values of 25% are considered as low-heterogeneity, 50% as moderate-heterogeneity, and 75% as high-heterogeneity [30]. Statistical significance was set at *p* ≤ 0.05. The effect sizes of the mean differences were estimated using Cohen’s criteria [32]. A small effect was conceptualized as *d* = 0.20, medium *d* = 0.50, and large *d* = 0.80.

Regarding the risk of AD and the cholesterol component, the direction of the reported effect size coefficient was reversed wherever necessary, such that all included effect sizes represented the association between cholesterol and an increase in the risk of suffering from AD, instead of a decrease in the AD risk. 

## 3. Results

A total of 331 studies were identified from major databases: 64 in ISI Web of Science (WOS), 141 in Scopus, 45 in PubMed, 79 in the Elsevier Science Direct and two in Google Scholar. 

**Figure 1:**
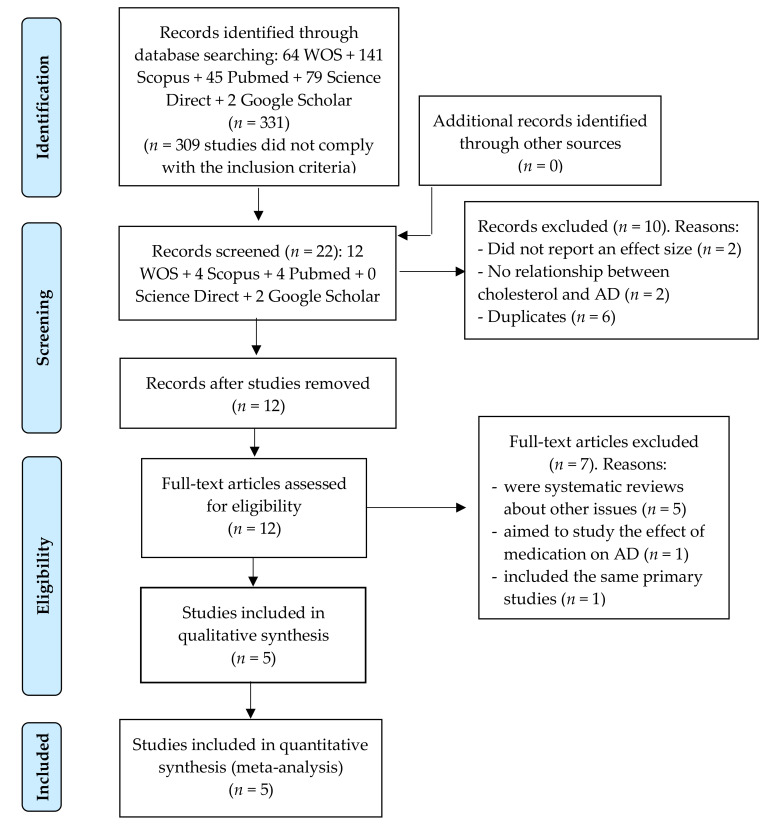
Flow chart depicting the selection of articles for our meta-meta-analysis.

Twenty-two meta-analyses were eligible for inclusion in this meta-meta-analysis. Of these, 17 were excluded because: (a) *k* = 2 did not report an effect size; (b) *k* = 2 did not provide information on the relationship between cholesterol and AD; (c) *k* = 6 were duplicated; (d) *k* = 5 were systematic reviews about other issues; (e) *k* = 1 aimed to study the effect of medication on AD; and (f) one meta-analysis that included the same primary studies as another study (see Figure 1). Finally, a total of *K* = 5 meta-analyses were analyzed in this meta-meta-analysis (*k* = 12 pooled effect sizes), including data from *n* = 100 primary studies (*n* = 236 effect sizes) (see Supplementary Table S1). 

Twelve effect sizes were extracted from a total of five meta-analyses. *K* = 3 effect sizes informed about LDL-C and risk of AD (25%); *k* = 3 about HDL-C (25%); *k* = 4 about TC (33.3%), and *k* = 2 of TG (16.7%). Table 1 summarizes the key features of the included primary diagnosis, design, number of primary studies, country of origin of the study, sample size, gender, mean age, results, total scores of quality of included meta-analyses (MAs) (AMSTAR) and effect sizes of the relationships between cholesterol and AD that were extracted. 

**Figure 2:**
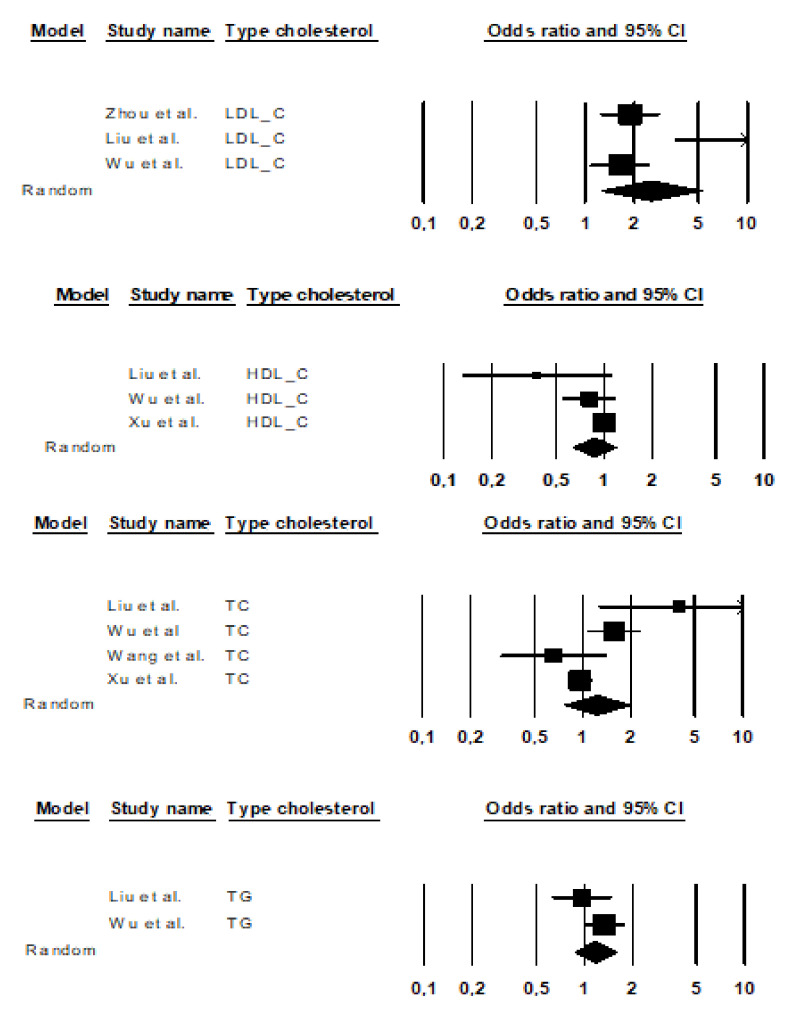
Forest plot of the effects of dyslipidemia on Alzheimer’s disease (AD): low-density lipoprotein cholesterol (LDL-C), high-density lipoprotein cholesterol (HDL-C), total cholesterol (TC), triglycerides (TG).

First, we investigated the relationship between overall cholesterol components and risk of AD in five meta-analyses, with a total of 2,289,511 participants, most of whom were female (*N* cases, AD = 19,757; *N* controls, HCs = 2,269,754). We identified a total of 12 estimates for cholesterol serum lipids (LDL-C, HDL-C, TC, and TG). The distribution of these estimates is shown in Figure 2. 

The total random effect of cholesterol on risk of AD was significant with *OR =* 1.29, 95% confidence interval (CI) [1.04, 1.60], *Z =* 2.28, *p =* 0.023, *d* = 0.14. When calculating the overall effect of lipid parameters, evidence of significant heterogeneity was found (*Q* = 45.49, *df =* 11, *p* = 0.0001, *I^2^* = 75.82%). Therefore, we examined whether subgroup analysis changed the results, as cholesterol levels at onset were significantly associated with AD. Heterogeneity could be explained, due to the different types of cholesterol: LDL-C, HDL-C, TG and TC. The results indicated that there were differences between the types of cholesterol: *Qb* = 9.04, *df* =3, *p* = 0.029. Hence, independent analyses for each type of cholesterol were performed. 

### 3.1. Studies of LDL-C Levels

The total random effect of LDL-C levels on AD was significant for *k* = 3 meta-analysis (*n* = 17,764, *n* = 5693 AD, and *n* = 12,071 HCs): *OR* = 2.55, 95% CI [1.25, 5.22], *Z =* 2.57, *p* = 0.010, *d* = 0.52. 

The first study conducted by Zhou et al. [33] provided information of *K* = 20 studies that compared serum LDL-C levels in AD and HC subjects (*N* = 7033 participants: 2266 AD and 4767 HCs). Liu et al. [1] also compared AD (*n* = 584 AD) and HC participants (*n* = 2130), examining *K*= 9 independent studies with an *N* = 2714. Finally, Wu et al. [12] informed about LDL-C, comparing *K*= 33 studies (*N* = 8017) with *n* = 2843 AD and *n* = 5174 HC participants. Results indicated that the LDL-C serum levels were significantly higher in AD patients than in HC subjects. Heterogeneity was significant (*Q* = 9.05, *df* = 2; *I*^2^ = 77.89%, *p* = 0.011, *I*^2^ = 77.89%). (See Table 2).

### 3.2. Studies on HDL-C Levels

Three meta-analyses *K* = 3 (*N* = 23,642, *n* = 4147 AD and *n* = 19,495 HCs) showed a non-significant effect of HDL-C levels on the risk of AD: *OR* = 0.87, CI 95% [0.64, 1.18], *Z* = −0.89, *p* = 0.372, *d* = 0.08 

Liu et al. [1] included 11 studies that analyzed HDL-C serum levels in AD patients and HCs. The combined sample size consisted of 2960 participants: 727 AD and 2233 HCs. They found non-significant differences between AD and HC subjects in HDL-C serum levels. Likewise, no differences were found between AD and HCs (*K* = 33 studies; *N* = 8192, *n* = 2921 AD and *n* = 5271 HCs) in the meta-analysis conducted by Wu et al. [12]. Finally, Xu et al. did not find any association between a lower level of HDL-C and AD (*K* = 6 studies; *N* = 12,490, *n* = 499 AD and *n* = 11,991 HCs). Heterogeneity was non-significant (*Q* = 3.85, *df* = 2; *I*^2^ = 47.98%, *p* = 0.146). (See Table 3).

### 3.3. Studies on TC Levels

Results indicated that *K* = 4 meta-analyses (*N* = 2,271,785, *n* = 16,704 AD and *n* = 2,255,081 HCs) informed about the TC and AD risk. The combined effect size showed that TC levels increased by 44% the risk of AD, but this effect did not reach statistical significance: *OR* = 1.44 CI 95% [0.91, 2.28], *Z* = 1.55, *p* = 0.121, *d* = 0.20.

Liu et al. [1] included *k* = 13 primary studies (*N* = 3112) that compared the TC serum levels in AD (*n* = 809) and HC subjects (*n* = 2303), showing that TC levels were significantly higher in AD patients than in HC participants. Likewise, Wu et al. [12] reviewed *K* = 33 studies (*N* = 7850, *n* = 2661 AD patients and *n* = 5189 HCs), finding significant effects. However, Wang et al. [18] evaluated total of *K*= 16 studies (*N* = 1653), including 959 subjects with AD and 694 controls, finding non-significant differences between AD and HDs. In this study, the authors analyzed the markers of cholesterol in subjects with AD with age-matched controls. Finally, Xu et al. [13], in a longitudinal study, also reported non-significant differences between AD and HCs in TC levels (*K* = 16 studies; *N* = 2,259,170, *n* = 12,275 AD and *n* = 2,246,895 HCs). Heterogeneity was significant (*Q* = 11.83, *df* = 3; *I*^2^ = 74.77%, *p* = 0.008). (See Table 4).

### 3.4. Studies of TG Levels

The combined effect size of studies of TG levels *K* = 2 (*N* = 8085, *n* = 2865 AD and *n* = 5220 HCs), *OR* = 1.22, CI 95% [0.96, 1.56], *Z* = 1.64, *p* = 0.102, *d* = 0.11, indicates that there was no significant association between overall TG and the risk of AD. Liu et al. [1] (*K* = 6; *N* = 512, *n* = 273 AD, and *n* = 239 HCs) and Wu et al. [12] (*K* = 28; *N* = 7573, *n* = 2592 AD and *n* = 4981 HCs) showed that there were no differences in TG serum levels between patients and controls. Heterogeneity analysis was non-significant *(Q =* 0.91, *df =* 1; *I^2^=* 0%, *p =* 0.340). (See Table 5).

## 4. Discussion

This study analyzes the association between cholesterol levels and the risk of developing AD. This is the first attempt to evaluate this relation by identifying previous meta-analyses and their primary studies analyzed worldwide. The present meta-meta-analysis summarizes the information of 100 primary studies and expands the findings of individual studies. 

Global results revealed that the level of cholesterol is a risk factor for AD. This finding is consistent with those from several prior studies, in which high cholesterol levels were associated with a higher likelihood of developing AD [1,4,12,13]. However, sensitivity analysis yielded several interesting and informative results. Even though the studies revealed that AD is involved in lipid metabolism, the results indicated that the effect of LDL-C, HDL-C, TC and TG on the development of dementia was different. We found that, compared with HC subjects, LDL-C levels were higher in AD participants, whereas HDL-C, TC and TG levels were not sensitive hallmarks of AD. 

An elevated LDL cholesterol level was an independent risk factor for the development of AD. The pooled effect size exhibited a significant increase in the risk of AD for individuals with higher levels of LDL-C. Other prospective studies also support these results, showing that LDL concentration in mid-life increases the risk of developing AD in later life [34]. Nevertheless, in this study, the pathways through which elevated LDL cholesterol levels influence the development of dementia are unclear [35].

First, previous research indicated that the senile plaques theories may provide a link between high LDL-C and AD [36]. In this theory, elevated levels of LDL-C and TC cause the extracellular deposition of amyloid protein (Aβ), hindering neuronal synaptic connections in the brain and increasing the risk of AD [37].

Second, the Tau protein may play an important role in proper axonal transport and overall neural integrity [38] and correlates with cognitive decline in the AD. In this case, cognitive loss is associated with an excess of the Tau protein, which causes neurofibrillary tangles and prevents the synaptic connection of neurons in the brain [39]. 

In addition, risk factors for vascular disease may also be risk factors for AD, and high blood LDL-C levels are vascular risk factors [40]. Indeed, various studies have demonstrated that high concentrations of LDL cholesterol are associated with coronary heart disease and carotid artery atherosclerosis, which, in turn, may lead to cognitive decline through cerebral embolism or hypoperfusion [41,42,43,44]. The study conducted by Moroney et al. [45] also demonstrated that the level of LDL cholesterol is a potential risk factor for dementia with stroke. Therefore, it is necessary to analyze the influence of other factors related to LDL-C in the development of AD. This result could explain the heterogeneity between LDL-C studies found in this meta-meta-analysis. 

The results showed no difference in HDL-C serum levels between AD and HC subjects. However, this result remains controversial, and no conclusive evidence was found. Various studies indicated that variations in HDL serum lipid levels are not associated with AD [1,12,13,35]. In other studies, lower levels of HDL have been associated with a high risk of AD [37,40]. Conversely, evidence suggests that high HDL-C levels are associated with a reduced risk of dementia, and that HDL may protect people against cerebrovascular dysfunction in AD [46]. In fact, cholesterol is an essential molecule for many physiologic processes and has multiple beneficial effects. Cholesterol is a precursor of steroid hormones (estrogens, androgens, vitamin D), it provides structural integrity and modulates the fluidity of cell membranes and is a main component of basic synaptic integrity and neurotransmission [47]. Moreover, HDL is known to have antioxidant and anti-inflammatory properties, which can affect neuroinflammatory responses in the brain and improve cognitive functions [48]. 

Whereas TC (total cholesterol) has been identified as a lipid marker for hyperlipemia [1,12,18], the summarized results did not find significant effects of TC levels on AD. Four meta-analyses assessing the effects of mid-life serum cholesterol on late-life risk of dementia and AD have yielded conflicting results. Several studies state that high cholesterol levels in middle age represent a risk factor for AD, but that there are no detectable differences in cholesterol levels at advanced ages [33,35,46]. Therefore, the non-significant effects of TC on AD in prospective studies (30 years to follow-up) could be explained by the variations in TC levels and the disease progression. Along these lines, Lepara et al. [44] indicated that cholesterol may be associated with AD cross-sectionally. In the same vein, Reitz [13] concluded that there is an association between higher cholesterol levels and a lower risk of AD, because of the nutritional status of elderly patients. In the early stages of AD, patients show alterations in the energy profile (weight loss, reduced caloric intake and increased energy requirements), and low cholesterol levels may reflect malnutrition [47]. Similarly, experimental studies and retrospective analyses in cohort studies indicate that statins could also affect the natural progress of the AD and reduce its prevalence over time [48]. Finally, even though Wang et al. [18] used a cross-sectional design, they did not find significant effects of TC on AD. In this study, the authors explained that cholesterol homeostasis could be altered in preclinical AD, whereas cholesterol dysregulation occurs throughout the disease’s process. This evidence could make it more difficult to find a significant relationship between TC and AD during the disease’s progress [18]. Hence, additional analysis is necessary.

The triglyceride serum level did not show a positive association with the development of AD in this meta-meta-analysis. This result also may be explained because of the retrospective design of some of the studies included herein. As we noted before, the use of cholesterol-lowering drugs could have suppressed the development of AD in participants, decreasing the likelihood of finding an association between TC and AD [49,50]. For instance, Wolozin [49] concluded that the use of statins, including lovastatin and pravastatin, decreased the development of AD. Other studies did not find that high triglyceride levels were associated with AD [1,12] and with potential changes in cognitive performance [51]. However, the results are not robust. Many studies associate hypercholesterolemia with the risk of dementia. Kivipelto [52] concluded that hypercholesterolemia could increase the risk of dementia, because arteriosclerosis occurs in the blood vessels, and this can alter blood flow, and directly induce neurodegeneration of AD [53]. Likewise, a recent study that investigated the association between diet and the level of triglycerides in the blood concluded that TG was associated with cognitive decline [54]. This result highlighted that a healthy diet and a good lifestyle for controlling the serum lipid levels was beneficial for preventing AD, which seems to counteract the scientific literature, where TG level is not associated with AD [55]. 

Our summary results showed no statistically significant differences between serum HDL-C, TC and TG levels in patients with AD compared with HC participants. Based on all available information, this study reveals that it is important to identify early risk factors for AD, because the neurodegenerative processes of AD can begin at an early age, and pharmacological and non-pharmacological therapies that delay the neurodegenerative progress of AD may be performed. Moreover, it may be necessary for future studies to investigate in more detail the neural regions that exhibit different cholesterol content regarding the pathological processes related to AD [56], and the influence of other potential moderators that could explain the heterogeneity between the primary results. Hence, the relevance of our findings for the pathophysiology of AD needs to be further explored in future research.

The limitations of this study include the possibility of misclassification bias when using single baseline measurements of cholesterol and the lack of verification of the clinical diagnosis of dementia subtype. We were also unable to investigate the effect of other moderator variables, such as country or cohort. Perhaps the relationship between lipid levels and the risk of probable AD would change if the same cohort were analyzed. Moreover, we could not assess the possible association between dietary and exercise levels and LDL-C, HDL-C, TC and TG serum levels. In addition, other variables have been associated with AD, but the meta-analysis included lacked a description of these factors, so the results could not be further adjusted. Body mass index, smoking status, stroke, hypertension, Type 2 diabetes and heart disease are also closely related to blood lipid levels, and could affect the risk of AD. 

However, this meta-meta-analysis represents a step toward evidence-based of AD and its relationship with dyslipidemia. First, this meta-meta-analysis provides an update and complete summary of the association of LDL-C, HDL-C, TC and TG with the prevalence of AD. Second, the effect sizes of one of the most studied risk factors for AD are provided to all healthcare professionals. Cholesterol is a modifiable risk factor, so if professionals know the relationship between cholesterol and AD, they could try to modify cholesterol levels to help to reduce AD risk. This study provides empirical evidence for the reduction of LDL-C levels through the promotion of healthy lifestyles (such as diet, weight control or physical activity) and/or the prescription of different medical treatments.

## 5. Conclusions

To sum up, the association of cholesterol and AD was evaluated. This meta-meta-analysis indicates that there is an association between the effect of cholesterol and AD. LDL-C, HDL-C, TC and TG were analyzed separately. LDL-C has a significant impact on the development of AD. Overall, this meta-meta-analysis represents a step toward evidence-based knowledge of AD. 

The understanding of risk factors and protective factors of AD would require more long-term studies, conducting exhaustive follow-ups of each patient. Furthermore, this study highlighted the need to analyze other factors related to AD. Indeed, physical activity and the use of drugs could reduce the effects of cholesterol on AD; hence, more research is necessary. This meta-meta-analysis provides more knowledge about the relationship between cholesterol and AD, which could have a huge beneficial impact on AD incidence and prevalence.

## Figures and Tables

**Table 1:** Population characteristics in studies of Alzheimer’s disease (AD)and cholesterol.

Study	Variable	Total *n*	Design	*K*	Country (*N*)	Sample	% F	Age	Result	Effect Size			AMSTAR Scores
										Effect Size	95% *CI* LL~UL	*p*	
Zhou et al. [33]	LDL-C	AD *n =* 2266 HC *n =* 4767	C	20	EU (7), USA (6), AS (4), AF (2), OC (1)	AD *n =* 2266 HC *n* = 4767	69.50	50-87	> LDL-C > AD	*SMD* = 0.35	0.12~0.58	<0.01	10
Liu et al. [1]	LDL-C	AD *n =* 891 HC *n* = 2399	C	9	EU (3), USA (4), AS (2)	AD *n* = 584 HC *n* = 2130	70	59-92	> LDL-C > AD	SMD = 1.40	0.70~2.10	0.000	11
	HDL-C			11	EU (4), USA (4), AS (3)	AD *n* = 727 HC *n* = 2233			HDL-C = AD	*SMD* = −0.53	−1.12~0.07	0.082	
	TC			13	EU (6), USA (4), AS (3)	AD *n* = 809 HC *n* = 2303			> TC > AD	*SMD* = 0.76	0.13~1.40	0.019	
	TG			6	EU (4), USA (2)	AD *n* = 273 HC *n* = 239			> TG = AD ns.	*SMD* = −0.02	−0.25~0.21	0.859	
Wu et al. [12]	LDL-C	AD *n* = 3037 HC *n* = 5375	C	33	AS (33)	AD *n* = 2843 HC *n* = 5174	53.87	56–84	> LDL-C > AD	*OR* = 1.64	1.07~2.51		10
	HDL-C			33		AD *n* = 2921 HC *n* = 5271			< HDL = AD ns.	*OR* = 0.81	0.55~1.19		
	TC			33		AD *n* = 2661 HC *n* = 5189			> TC > AD	*OR* = 1.58	1.10~2.92		
	TG			28		AD *n* = 2556 HC *n* = 4903			> TG = AD ns.	*OR* = 1.33	0.99~1.79		
Wang et al. [18]	TC	AD *n* = 959 HC *n* = 694	C	16	-	AD *n* = 959 HC *n* = 694	60.21	60–94, *M* = 71.38	> TC = AD	*SMD* = −0.23	0.65~0.19	0.29	10
Xu et al. [13]	HDL-C	AD *n* = 12604 HC *n* = 2,256,519	L(2–9)	6	USA (2), EU (4)	AD *n* = 499 HC *n* = 11,991	56.3	*M* = 71.21	> HDL = AD	*RR* = 1.00	0.86~1.14	0.942	11
	TC		L (3.2–32)	16	USA (8), EU (4), AS (4)	AD *n* = 12275 HC *n* = 2,246,750	49.5	*M* = 68.5	> TC = AD	*RR* = 0.96	0.81–1.11	0.000	

Note: Variables: AD: Alzheimer’s disease; LDL-C: low-density level cholesterol; HDL-C: high-density level cholesterol; TC: total cholesterol; TG: triglycerides; Total N of each study; Design: C: cross-sectional; L: longitudinal (year); *K*: number of studies; Country: *N*: number of independent studies. EU: European Union; USA: United States of America; AS: Asia; AF: Africa; OC: Oceania; ^6^ Independent Sample: AD: Alzheimer´s disease cases; HC: healthy control participants for each type of cholesterol.; F: females; M: mean; *CI*: 95% confidence interval; *SMD*: standard mean difference; *OR*: odds ratio; *RR*: risk ratio.

**Table 2:** Summary effect sizes for low-density lipid cholesterol (LDL-C) serum levels and Alzheimer’s disease (AD).

Model	Study	Statistics						
		*OR*	Lower Limit	Upper Limit	*Z*	*p*	Weight (Random)	Std Residual
	Zhou et al. [33]	1.89	1.24	2.86	2.98	0.003	40.64	−0.69
	Liu et al. [1]	12.67	3.56	45.08	3.92	0.000	18.96	2.13
	Wu et al. [12]	1.64	1.07	2.50	2.25	0.024	40.39	−1.01
Random effect		2.55	1.25	5.22	2.57	0.010		

**Table 3:** Summary effect sizes for high-density lipid cholesterol (HDL-C) serum levels and AD.

Model	Study	Statistics						
		*OR*	Lower Limit	Upper Limit	*Z*	*p*	Weight (Random)	Std Residual
	Liu et al. [1]	0.38	0.13	1.13	−1.75	0.081	7.35	−1.46
	Wu et al. [12]	0.81	0.55	1.19	−1.07	0.285	33.30	−0.31
	Xu et al. [13]	1.00	0.87	1.16	0.00	1.000	59.35	1.08
Random effect		0.87	0.64	1.18	−0.89	0.374		

**Table 4:** Summary effect sizes for total cholesterol (TC) serum levels and Alzheimer’s disease (AD).

Model	Study	Statistics						
		*OR*	Lower Limit	Upper Limit	*Z*	*p*	Weight (Random)	Std Residual
	Liu et al. [1]	3.97	1.25	12.55	2.35	0.019	11.41	1.55
	Wu et al. [12]	1.57	1.09	2.28	2.39	0.017	31.52	0.26
	Wang et al. [18]	1.52	0.70	3.25	1.07	0.283	19.04	0.11
	Xu et al. [13]	0.96	0.83	1.12	−0.4 −0.53	0.597	37.25	−1.35
Random effect		1.44	0.91	2.28	1.55	0.121		

**Table 5:** Summary effect sizes for triglycerides (TG) serum levels and Alzheimer’s disease (AD).

Model	Study	Statistics						
		*OR*	Lower Limit	Upper Limit	*Z*	*p*	Weight (Random)	Std Residual
	Liu et al. [1]	1.04	0.68	1.57	0.17	0.864	33.61	−0.96
	Wu et al. [12]	1.33	0.99	1.79	1.89	0.059	66.39	0.96
Random effect		1.22	0.96	1.56	1.64	0. 102		

## Supplementary Materials

The following are available online at https://www.mdpi.com/2076-3425/10/6/386/s1. Table S1 showed the available primary studies of cholesterol and AD (*K* = 100 studies) and the main characteristics. It is worth noting that the search for suitable meta-analyses was systematic. To carry out the main analysis, cholesterol studies were divided into groups based on the type of lipid serum at which cholesterol was placed in each meta-analysis: LDL-C, HDL-C, TC, and TG. Table S2 illustrates the individual effect sizes obtained from the meta-analysis of the 100 primary studies to facilitate the replicability of this study and further analysis.
