# Supplementary material for: Cholesterol and Alzheimer’s Disease Risk: A Meta-Meta-Analysis
Source: Brain Sci. 2020 Jun 18;10(6):386. doi: 10.3390/brainsci10060386 (PMC7349210; doi:10.3390/brainsci10060386)
Supplement: Supplementary file 1 [file brainsci-10-00386-s001.pdf]

Supplementary material

Table 1. Characteristics of studies included in the meta-analysis

			Cases			Controls		
**Study (*N* = 100)**	**Year**	**Country**	***n***	**% F**	**Age**	***n***	**% F**	**Age**
**Zhou et al.** [33]								
1. Ban et al. [57] *	2009	Japan	197	59.90	80 ± 1	47	38.30	75 ± 1
2. Cacabelos et al. [58]	2003	Spain	147	-	71.73 ± 9.61	109	-	50.20 ± 12.06
3. Caramelli et al.[59]	1999	Brazil	24	-	67.2 ± 10.6	32	59.38	68.2 ± 10.6
4. Chen et al. [60]	2019	China	117	52.14	67.64 ± 6.65	117	62.39	66.06 ± 6.00
5. Hoshino, Kamino, Matsumoto [61] *	2002	Japan	82	71.95	77.0 ± 6.8	40	67.50	84.2 ± 3.1
6. Kouzuki et al. [62]	2018	Japan	42	61.90	80.5 ± 5.7	18	72.22	75.6 ± 5.5
7. Kuo et al. [63] *	1998	America	64	-	81.6 ± 0.9	36	-	78.7 ± 1.3
8. Lehtonen, Luutonen [64]	1986	Finland	22	100.00	≥90	23	100.00	≥90
9. Lesser et al. [65]	2001	America	44	-	87.0 ± 8.5	22	-	82.0 ± 7
10. Macesic et al. [66]	2017	Serbia	62	70.97	73.1 ± 5.8	40	50.00	68.4 ± 5.5
11. Mamo et al. [67]	2008	Australia	10	-	79.2 ± 1.8	10	-	80.5 ± 1.5
12. Moroney et al. [45]	1999	America	225	72.00	77.7 ± 6.3	764	67.54	74.1 ± 5.5
13. Panza et al. [68]	2003	Italy	49	69.39	71.6 ± 9.3	45	71.11	65.8 ± 11.6
14. Paragh et al. [69]	2002	Hungary	30	66.67	64.3 ± 11.7	40	65.00	72.3 ± 9.6
15. Reitz et al. [37]	2004	America	244	77.46	82.85 ± 7.3	2226	65.86	76.42 ± 6.3
16. Ryglewicz et al. [70]	2002	Poland	26	-	67 ± 8.4	46	-	67.5 ± 6.9
17. Scacchi et al. [71]	1998	Italy	80	71.25	83.5 ± 5.9	155	76.77	78.3 ± 7.0
18. Shafagoj et al. [72]	2018	Jordan	38	63.16	74.2 ± 5.4	33	66.67	72.4 ± 6.3
19. Solfrizzi et al. [73] *	2002	Italy	49	75.51	71.6 ± 9.3	45	71.11	65.8 ± 11.6
20. Tang et al. [74]	2019	China	143	45.45	62.89 ± 8.38	140	46.43	64.10 ± 9.49
21. Warren, Hynan, Weiner [75]	2012	America	150	70.00	79.5 ± 6.17	197	69.04	70 ± 6.33
22. Watanabe et al. [76] *	2005	Japan	106	-	79 ± 7	227	-	76 ± 10
23. Werh et al. [77]	2006	Sweden	97	90.72	77.9 ± 3.0	139	94.24	78.5 ± 3.0
24. Wolf et al. [78]	2004	Japan	25	4.00	80 ± 6	26	34.62	77 ± 5
25. Yamamoto et al. [79]	2005	Turkey	61	19.67	74.1 ± 7.4	32	38.46	74.5 ± 6.3
26. Yavuz et al. [80]	2008	Poland	132	75.00	71.8 ± 7.9	158	58.86	70.5 ± 8.8
**Liu et al. [1]**								
Lehtonen, Luutonen [64]*	1986	Finland	22	-	>90	23	-	>90
Kuo et al. [63]	1998	USA	64	-	81.6	36	-	78.7
27. Kalman et al. [81]	1999	USA	24	75.00	70.2	15	73.00	64.8
28. Merched et al. [82]	2000	France	98	71.4	77.56	59	52.5	75.37
Solfrizzi et al. [73]	2002	Italy	18	0.00	74.2	30	0.00	68.4
Solfrizzi et al. [73]	2002	Italy	43	100.00	70.2	33	100.00	67
29. Lesser et al. [83][1]	2009	USA	144	-	-	151	-	-
30. Sun et al. [84]	2010	China	45	64.00	59-92	44	68.00	58-87

Table 1. Characteristics of studies included in the meta-analysis

			Cases			Controls		
**Study (*N* = 100)**	**Year**	**Country**	***n***	**% F**	**Age**	***n***	**% F**	**Age**
31. Preseki et al. [85]	2011	Croatia	50	-	79.1	58	-	71.6
32. Parnowski, Kaluza # [86]	2013	Poland	39	66.6	80.12	44	75.00	72.95
33. R V ~	2016	India	167	0.00	69.8	984	0.00	63.1
R V ~	2016	India	137	100.00	67.8	882	100.00	62.4
34. Grossi et al. [87]	2018	Brazi	40	30.00	78(7)	40	31.00	76.5
**Wu et al.** [12]								
35. Agarwal et al. [88]	2015	Indian	39	69	67±9	42	52	62±8
36. Alam et al. [89]	2014	Indian	75	68	66±9	120	63	63±8
Ban et al. [57]	2009	Japanese	197	40	80±1	47	62	75±1
37. Cankurtaran et al. [90]	2005	Turks	120	34	74±8	803	37	71±6
38. Chang et al. [91]	2014	Chinese	44	/	80±9	62	NA	80±8
39. Duan, Ling, Zhou [92]	2006	Chinese	62	47	65±3	50	60	56±3
40. Han [93]	2005	Chinese	27	33	72±11	27	NA	NA
Hoshino, Kamino, Matsumoto [61]	2002	Japanese	82	28	84±3	40	33	/77±7
41. Li [94]	2014	Chinese	45	36	64±2	45	36	64±3
42. Liu, Chen [95]	2006	Chinese	31	45	69±7	40	43	69±8
43. Liu [96]	2005	Chinese	268	47	74±3	325	46	74±2
44. Raygani et al. [97]	2006	Iranians	94	44	74±10	111	37	72±11
45. Shim [98]	2010	Korean	78	40	72±9	58	36	63±7
46. Singh et al. [99]	2012	Indian	70	76	NA	75	NA	NA
47. Sun [100]	2006	Chinese	82	55	81±3	46	83	78±4
48. Vasantharekha et al. [101]	2016	Indian	304	47	69±5	1868	53	63±2
49. Wada [102]	2000	Japanese	36	29	77±5	15	13	72±6
50. Wang et al. [103]	2005	Chinese	35	35	69±8	16	44	70±7
51. Wang [104]	2006	Chinese	124	47	NA	80	50	NA
52. Wang [105]	2017	Chinese	43	44	67±10	45	36	64±6
53. Wang, Zhang [106]	2016	Chinese	39	41	68±7	40	45	71±7
Wang, Zhang [106]	2016	Chinese	34	/	74±8	40	45	71±7
54. Watanabe et al. [107]	2004	Japanese	34	76	76±9	63	NA	72±11
Watanabe et al. [76]	2005	Japanese	106	55	79±7	227	70	76±10
55. Xiao et al. [108]	2012	Chinese	104	36	78±7	104	54	77±6
56. Xiao hong et al. [109]	2010	Chinese	45	39	59 ~ 92	44	32	58 ~ 87
Yamamoto et al. [79]	2005	Japanese	61	33	80±6	32	53	77±5
57. Yang, Tian, Zhong [110]	2007	Chinese	15	45	73±8	29	56	60±7
58. Yu et al. [111]	2014	Chinese	201	33	77±6	257	47	76±7
59. Yuan et al. [112]	2006	Chinese	30	38	71±7	60	30	65±6
60. Yue et al. [113]	2009	Chinese	111	48	69±9	117	38	66±9
61. Zengi et al. [114]	2012	Turks	21	50	76±8	20	55	81±7
62. Zhao et al. [115]	2014	Chinese	48	33	69±6	37	51	71±6
63. Zheng et al. [116]	2016	Chinese	207	50	81±8	256	35	82±6
64. Zhong et al. [117]	2016	Chinese	54	55	70±8	54	54	71±7
65. Zhou [118]	2015	Chinese	40	45	68±2	40	58	68±3

Table 1. Characteristics of studies included in the meta-analysis

			Cases			Controls		
**Study (*N* = 100)**	**Year**	**Country**	***n***	**% F**	**Age**	***n***	**% F**	**Age**
66. Zhu [119]	2007	Chinese	31	43	69±7	40	43	69±7
**Wang et al.** [18]								
67. Papassotiropoulos et al. [120]	2002		32	62.00	69 ± 8	7	29.00	55 ± 10
68. Martínez-Morillo et al. [121]	2014		38	64.00	(60-94)	37	53.00	(43-80)
69. Kölsch et al. [122]	2006		75	63.40	68.4 ± 7.9	39	53.80	65.9 ± 11.4
70. Kölsch et al. a [123]	2009		118	61.90	68.5 ± 7.9	62	57.90	70.4 ± 7.1
71. Kölsch et al. b [124]	2009		149	69.10	74.1 ± 7.9	86	53.50	72.8 ± 7.6
72. Kölsch et al. [125]	2010		90	63.90	70.6 ± 8.3	57	51.60	69.3 ± 6.8
73. Qureischie et al. [126]	2008		104	68.00	72.5 ± 8.8	49	72.40	72.4 ± 7.9
74. Mateos et al. [127]	2011		21	66.70	67.3 ± 1.70	28	67.90	57.8 ± 1.27
75. Wollmer et al. [128]	2003		24	58.00	73.5 ± 5.5	22	49.40	70.1 ± 6.3
76. Wollmer et al. [129]	2003		24	-	71.7 ± 7.8	22	-	65.6
77. Shafaati et al. [130]	2007		17	41.20	(62–83)	43	65.10	(18–85)
78. Schönknecht et al. [131]	2002		17	42.90	75.4 ± 10.3	55	40.00	69.0 ± 5.8
79. Popp et al. [132]	2012		53	62.30	71.23 ± 8.29	43	51.20	67.33 ± 9.04
80. Popp et al. [133]	2013		106	64.20	71.1 ± 7.87	87	49.40	67.7 ± 9.13
81. Vanmierlo et al. [134]	2011		67	44.80	71.8 ± 7.5	29	62.70	69.0 ± 6.9
82. Leoni, Caccia [135]	2013		24	70.80	66.8 ± 8.0	28	65.80	68.6 ± 2.85
**Xu et al.** [13]								
83. Tan et al. [35]	2003	USA	77	-	-	1026	62.96	76.1
84. Li et al. [136]	2005	USA	152	-	-	2123	59.20	74.9
85. Solomon et al. [137]	2009	USA	469	59.91	69.90	9844	0	-
86. Mielke et al. [138]	2010	Sweden	46	-	-	648	0	-
87. Huang et al. [139]	20s14	Taiwan	612	-	-	142744	48.20	-
88. Kivipelto et al. [140]	2005	Karelia	48	-	-	1449	63.14	-
89. Reitz et al. [48]	2010	USA	101	65.34	79.70	1130	65.66	75.7
90. Kimm et al. [141]	2011	Korea	821	-	-	490445	0	51.9
Kimm et al. [141]	2011	Korea	1030	100	-	358060	100	53.6
91. Kivipelto et al. [142]	2002	Finland	48	-	-	1449	66.04	-
92. Hayden et al. [143]	2006	UK	104	73.07	81.50	3264	58.24	74
93. Notkola et al. [144]	1998	Finland	27	-	-	444	0	-
94. Rönnemaa et al. [145]	2012	Sweden	127	-	-	2268	-	49.6
95. Wang et al. [146]	2012	Taiwan	8488	-	-	1230400	51.95	-

Table 1. Characteristics of studies included in the meta-analysis

			Cases			Controls		
**Study (*N* = 100)**	**Year**	**Country**	***n***	**% F**	**Age**	***n***	**% F**	**Age**
96. Lieb et al. [147]	2012	USA	18	100	73.97	99	100	-
97. Dal Forno et al. [148]	2005	USA	40	-	-	576	0	66.8
Dal Forno et al. [148]	2005	USA	67	100	-	781	100	64
Singh et al. [99]	2012	India	0	-	-	0	-	-
98. Forti et al. [149]	2010	Italy	18	-	-	466	51.28	69.3
Forti et al. [149]	2010	Italy	30	-	-	238	67.64	79.8
99. Raffaitin et al. [150]	2009	France	134	-	-	7087	60.99	-
100. Muller et al. [151]	2007	USA	147	-	-	1833	67.32	76.1
Singh et al. [99]	2012	India	0	-	-	145	-	-

**K*=6 articles were duplicated in LDL-C. The number of participants were deleted for main analysis.

[1] Total *N* Cases = 144; n/LDL-C=41; n/HDL-C=41; n/TC=62; Total *N* Control = 151; n/LDL-C=48; n/HDL-C=48; n/TC=55

Table 2. Factors included in primary meta-analysis that showed positive and negative association with AD.

Study	LDL_C		HDL_C		TC		TG	
	SMD (95% CI)	Weight						
**Zhou et al.** [33]								
Ban et al. [57]	0.80 (0.47 ~ 1.12)	4.1	-	-	-	-	-	-
Cacabelos et al. [58]	0.01 (-0.24 ~ 0.26	4.2	-	-	-	-	-	-
Caramelli et al. [59]	0.14 (-0.39 ~ 0.67)	3.6	-	-	-	-	-	-
Chen et al. [60]	1.19 (0.91 ~ 1.47)	4.2	-	-	-	-	-	-
Hoshino, Kamino, Matsumoto [61]	0.34 (-0.04 ~ 0.72)	3.9	-	-	-	-	-	-
Kouzuki et al. [62]	-0.22 (-0.77 ~ 0.34)	3.5	-	-	-	-	-	-
Kuo et al. [63]	4.45 (3.70 ~ 5.20)	3.0	-	-	-	-	-	-
Lehtonen, Luutonen [64]	0.57 (-0.03 ~ 1.17)	3.4	-	-	-	-	-	-
Lesser et al. [65]	0.32 (-0.19 ~ 0.84)	3.6	-	-	-	-	-	-
Macesic et al. [66]	1.07 (0.65 ~ 1.50)	3.8	-	-	-	-	-	-
Mamo et al. [67]	-0.16 (-1.04 ~ 0.72)	2.7	-	-	-	-	-	-
Moroney et al. [45]	-0.25 (-0.40 ~ -0.10)	4.3	-	-	-	-	-	-
Panza et al. [68]	-0.63 (-1.04 ~ -0.21)	3.9	-	-	-	-	-	-
Paragh et al. [69]	2.04 (1.46 ~ 2.63)	3.4	-	-	-	-	-	-
Reitz et al. [37]	-0.00 (-0.13 ~ 0.13)	4.4	-	-	-	-	-	-
Ryglewicz et al. [70]	0.29 (-0.20 ~ 0.77)	3.7	-	-	-	-	-	-
Scacchi et al. [71]	-0.45 (-0.73 ~ -0.18)	4.2	-	-	-	-	-	-
Shafagoj et al. [72]	-0.32 (-0.79 ~ 0.15)	3.7	-	-	-	-	-	-
Solfrizzi et al. [73]	-0.69 (-1.11 ~ -0.28)	3.9	-	-	-	-	-	-
Tang et al. [74]	0.39 (0.15 ~ 0.62)	4.2	-	-	-	-	-	-
Warren, Hynan, Weiner [75]	0.50 (0.28 ~ 0. 72)	4.3	-	-	-	-	-	-
Watanabe et al. [76]	0.17 (-0.06 ~ 0.40)	4.2	-	-	-	-	-	-

Table 2. Factors included in primary meta-analysis that showed positive and negative association with AD.

Study	LDL_C		HDL_C		TC		TG	
Werh et al. [77]	0.36 (0.10 ~ 0.62)	4.2	-	-	-	-	-	-
Wolf et al. [78]	0.20 (-0.35 ~ 0.75)	3.50	-	-	-	-	-	-
Yamamoto et al. [79]	0.08 (-0.35 ~ 0.51)	3.80	-	-	-	-	-	-
Yavuz et al. [80]	-0.02 (-0.25 ~ -0.21)	4.20	-	-	-	-	-	-
**Liu et al. [1]**	***OR (95% CI)***	**Weight**	***SMD (95% CI)***	**Weight**	***SMD (95% CI)***	**Weight**	***SMD (95% CI)***	**Weight**
Lehtomen, Luutonen [64]	0.58 (-0.02 ~ 1.18)	10.86	-0.02 (-0.61-0.56)	8.77	0.43 (-0.16 ~ 1.02)	7.53	0.19 (-0.40 ~ 0.77)	6.32
Kuo et al. [63]	4.49 (3.74 ~ 5.24)	10.36	-2.76 (-3.33 ~ -2.20)	8.82	0.00 (-0.65 ~ 0.65)	7.54		-
Kalman et al. [81]	-0.19 (-0.83 ~ 0.46)	10.71	0.75 (0.08 ~ 1.42)	8.54	-0.40 (-0.72 ~ -0.07)	7.44	-0.31 (-0.96 ~ 0.34)	5.77
Merched et al. [82]	-	-	-1 (-1.34 ~ -0.66)	1.29	-1.15 (-1.78 ~ -0.52)	7.89	-0.26 (-0.5 ~ 0.07)	8.95
Solfrizzi et al. [73]	-	-	-	-	-0.17 (-0.62 ~ 0.28)	7.46	-	-
Solfrizzi et al. [73]	-	-	-	-	4.83 (4.11 ~ 5.55)	7.73	-	-
Lesser et al. [83]	3.26 (2.62 ~ 3.90)	10.73	1.38 (0.92 ~ 1.85)	9.05	0.57 (0.15 ~ 1)	7.29	-	-
Sun et al. [84]	-	-	-0.29 (-0.71 ~ 0.13)	9.15	0.45 (0.07 ~ 0.84)	7.77	-	-
Preseki et al. [85]	0.43 (0.05 ~ 0.81)	11.39	0.48 (80.10 ~ 0.87)	9.21	-0.31 (-0.74 ~ 0.13)	7.82	0.54 (0.15 ~ 0.92)	8.32
Parnowski, Kaluza # [86]	0.45 (0.01 ~ 0.89)	11.27	-1.04 (-1.50 ~ -0.58)	9.06	1.23 (1.05 ~ 1.40)	7.76	0.06 (-0.50 ~ 0.62)	6.57
R V	1.30 (1.12 ~ 1.47)	11.71	-1.50 (-1.67 ~ -1.32)	9.51	1.87 (1.67 ~ 2.07)	8.01	-	-
R V	2.26 (2.05 ~ 2.46)	11.68	-1.51 (-1.70 ~ -1.32)	9.49	-0.28 (-0.72 ~ 0.16)	7.99	-	-
Grossi et al. [87]	0.25 (-0.19-0.69)	11.27	-0.16 (-0.60 ~ 0.28)	9.11	0.76 (0.13 ~ 1.40)	7.75	-0.75(-1.20 ~ -0.30)	7.61
**Wu el al.** [12]	*SMD (95% CI)*				*SMD (95% CI)*			-
Agarwal et al. [88]	0.13 (-0.31 ~ 0.56)	-	0.13 (-0.31 ~ 0.56)	-	0.28 (-0.16 ~ 0.72)	-	-	-
Alam et al. [89]	0.15 (-0.14 ~ 0.44)	-	-0.22 (-0.51 ~ 0.07)	-	0.12 (-0.17 ~ 0.41)	-	0.18 (-0.11 ~ 0.46)	-
Ban et al. [57]	0.80 (0.47 ~ 1.12)	-	1.26 (0.92 ~ 1.60)	-	-	-	-1.25 (-1.58 ~ -0.91)	-
Cankurtaran et al. [90]	-0.02 (-0.21 ~ 0.17)	-	0.08 (-0.11 ~ 0.27)	-	0.04 (-0.15 ~ 0.23)	-	0.03 (-0.16 ~ 0.22)	-
Chang et al. [91]	-	-	0.11 (-0.27 ~ 0.50)	-	0.15 (-0.23 ~ 0.54)	-	-0.08 (-0.46 ~ 0.31)	-

Table 2. Factors included in primary meta-analysis that showed positive and negative association with AD.

Study	LDL_C		HDL_C		TC		TG	
Duan, Ling, Zhou [92]	-	-	-	-	0.82 (0.43 ~ 1.21)	-	0.78 (0.40 ~ 1.17)	-
Han [93]	0.00 (-0.53 ~ 0.53)	-	0.00 (-0.53 ~ 0.53)	-	0.00 (-0.53 ~ 0.53)	-	0.00 (-0.53 ~ 0.53)	-
Hoshino, Kamino, Matsumoto [61]	0.34 (-0.04 ~ 0.72)	-	-0.08 (-0.46 ~ 0.30)	-		-	-	-
Li [94]	-0.01 (-0.42 ~ 0.40)	-	-0.07 (-0.48 ~ 0.34)	-	0.00 (-0.41 ~ 0.41)	-	-	-
Liu, Chen [95]	0.85 (0.36 ~ 0.69)	-	0.05 (-0.42 ~ 0.52)	-	0.79 (0.30 ~ 1.28)	-	0.91 (0.42 ~ 1.40)	-
Liu [96]	0.52 (0.36 ~ 1.34)	-	-0.09 (-0.25 ~ 0.08)	-	0.33 (0.16 ~ 0.49)	-	0.06 (-0.10 ~ 0.23)	-
Raygani et al. [97]	0.52 (0.24 ~ 0.80)	-	-0.74 (-1.02 ~ -0.45)	-	0.38 (0.10 ~ 0.65)	-	0.17 (-0.11 ~ 0.45)	-
Shim [98]	-0.29 (-0.64 ~ 0.05)	-	0.16 (-0.18 ~ 0.50)	-	-0.28 (-0.62 ~ 0.06)	-	-	-
Singh et al. [99]	0.95 (0.61 ~ 1.30)	-	-0.54 (-0.87 ~ -0.21)	-	1.38 (1.02 ~ 1.74)	-	0.00 (-0.33 ~ 0.32)	-
Sun [100]	-0.29 (-0.65 ~ 0.07)	-	0.03 (-0.33 ~ 0.39)	-	-0.11 (-0.47 ~ 0.26)	-	0.14 (-0.22 ~ 0.50)	-
Vasantharekha et al. [101]	1.72 (1.59 ~ 1.85)	-	-1.48 (-1.61 ~ -1.36)	-	1.50 (1.37 ~ 1.63)	-	0.17 (0.05 ~ 0.29)	-
Wada [102]	-0.14 (-0.74 ~ 0.47)	-	0.25 (-0.36 ~ 0.85)	-	-0.37 (-0.98 ~ 0.24)	-	-0.58 (-1.19 ~ 0.04)	-
Wang et al. [103]	0.54 (-0.06 ~ 1.14)	-	0.14 (-0.45 ~ 0.73)	-	0.61 (0.01 ~ 1.22)	-	0.82 (0.21 ~ 1.43)	-
Wang [104]	1.62 (1.29 ~ 1.94)	-	-0.23 (-0.51 ~ 0.06)	-	-0.20 (-0.49 ~ 0.08)	-	1.36 (1.05 ~ 1.67)	-
Wang et al. [105]	-	-	-0.94 (-1.38 ~ -0.50)	-	-	-	-	-
Wang, Zhang [106]	0.00 (-0.44 ~ 0.44)	-	0.00 (-0.44 ~ 0.44)	-	0.00 (-0.44 ~ 0.44)	-	-	-
Wang, Zhang [106]	0.25 (-0.21 ~ 0.71)	-	0.00 (-0.46 ~ 0.46)	-	0.15 (-0.31 ~ 0.61)	-	-	-
Watanabe et al. [107]	0.03 (-0.39 ~ 0.45)	-	0.00 (-0.42 ~ 0.42)	-	-0.27 (-0.69 ~ 0.15)	-	-0.34 (-0.76 ~ 0.09)	-
Watanabe et al. [76]	0.17 (-0.06 ~ 0.40)	-	0.32 (0.09 ~ 0.55)	-	-0.08 (-0.31 ~ 0.16)	-	-0.27 (-0.50 ~ -0.04)	-
Xiao et al. [108]	0.08 (-0.19 ~ 0.35)	-	-0.36 (-0.63 ~ 0.08)	-	0.39 (0.12 ~ 0.67)	-	0.06 (-0.21 ~ 0.33)	-
Xiao hong et al., [109]	-	-	-0.73 (-1.16 ~ -0.30)	-	0.57 (0.15 ~ 1.00)	-	-	-
Yamamoto et al. [79]	0.08 (-0.35 ~ 0.51)	-	0.06 (-0.37 ~ 0.49)	-	-0.07 (-0.50 ~ 0.35)	-	-0.22 (-0.65 ~ 0.21)	-
Yang, Tian, Zhong [110]	-0.40 (-1.03 ~ 0.23)	-	0.55 (-0.09 ~ 1.18)	-	-0.08 (-0.71 ~ 0.54)	-	-0.19 (-0.81 ~ 0.44)	-
Yu et al. [111]	0.13 (-0.06 ~ 0.31)	-	-0.02 (-0.20 ~ 0.17)	-	0.30 (0.12 ~ 0.49)	-	-0.10 (-0.29 ~ 0.08)	-
Yuan et al. [112]	-1.06 (-1.53 ~ -0.60)	-	-0.06 (-0.50 ~ 0.38)	-	0.81 (0.35 ~ 1.26)	-	0.66 (0.22 ~ 1.11)	-

Table 2. Factors included in primary meta-analysis that showed positive and negative association with AD.

Study	LDL_C		HDL_C		TC		TG	
Yue et al. [113]	0.14 (-0.12 ~ 0.40)	-	-0.11 (-0.37 ~ 0.15)	-	0.31 (0.05 ~ 0.57)	-	0.46 (0.19 ~ 0.72)	-
Zengi et al. [114]	-0.57 (-1.19 ~ 0.06)	-	-0.26 (-0.88 ~ 0.35)	-	-0.87 (-1.51 ~ -0.23)	-	-1.48 (-2.17 ~ -0.79)	-
Zhao et al. [115]	0.05 (-0.38 ~ 0.48)	-	-0.96 (-1.42 ~ -0.51)	-	-0.26 (-0.69 ~ 0.18)	-	0.85 (0.40 ~ 1.30)	-
Zheng et al. [116]	0.08 (-0.10 ~ 0.27)	-	-0.21 (-0.39 ~ -0.03)	-	0.16 (-0.02 ~ 0.35)	-	0.33 (0.15 ~ 0.52)	-
Zhong et al. [117]	0.58 (0.19 ~ 1.30)	-		-		-	0.79 (0.40 ~ 1.18)	-
Zhou [118]	0.84 (0.39 ~ 1.30)	-	0.10 (-0.34 ~ 0.54)	-	0.78 (0.32 ~ 1.23)	-	-	-
Zhu [119]	0.85 (0.36 ~ 1.34)	-	0.05 (-0.42 ~ 0.52)	-	0.79 (0.30 ~ 1.28)	-	0.91 (0.42 ~ 1.40)	-
**Wang et al.** [18]					***SMD (95% CI)***			-
Papassotiropoulos et al. [120]	-	-	-	-	0.68 (-0.15 ~ 1.52)	-	-	-
Martínez-Morillo et al. [121]	-	-	-	-	0.06 (-0.40 ~ 0.51)	-	-	-
Kölsch et al. [122]	-	-	-	-	-2.78 (-3.31 ~ -2.25)	-	-	-
Kölsch et al. a [123]	-	-	-	-	-0.91 (-1.23 ~ -0.58)	-	-	-
Kölsch et al. b [124]	-	-	-	-	-0.62 (-0.89 ~ -0.35)	-	-	-
Kölsch et al. [125]	-	-	-	-	-0.45 (-0.78 ~ -0.11)	-	-	-
Qureischie et al. [126]	-	-	-	-	-1.28 (-1.65 ~ -0.91)	-	-	-
Mateos et al. [127]	-	-	-	-	0.40 (-0.17 ~ 0.97)	-	-	-
Wollmer et al. [128]	-	-	-	-	-0.72 (-1.31 ~ -0.12)	-	-	-
Wollmer et al. [129]	-	-	-	-	-0.67 (-1.26 ~ -0.07)	-	-	-
Shafaati et al. [130]	-	-	-	-	2.68 (1.93 ~ 3.43)	-	-	-
Schönknecht et al. [131]	-	-	-	-	0.44 (-0.11 ~ 0.99)	-	-	-
Popp et al. [132]	-	-	-	-	-0.37 (-0.77 ~ 0.04)	-	-	-
Popp et al. [133]	-	-	-	-	-0.42 (-0.71 ~ -0.13)	-	-	-
Vanmierlo et al. [134]	-	-	-	-	-0.30 (-0.74 ~ 0.14)	-	-	-
Leoni, Caccia [135]	-	-	-	-	1.10 (0.52 ~ 1.69)	-	-	-
**Xu et al.** [13]			***RR (95% CI)***	**Weight**	***RR (95% CI)***	**Weight**		-

Table 2. Factors included in primary meta-analysis that showed positive and negative association with AD.

Study	LDL_C		HDL_C		TC		TG	
Tan et al. [35]	-	-	-	-	0.97 (0.90 ~ 1.05)	16.01	-	-
Li et al. [136]	-	-	1.23 (0.71 ~ 2.15)	3.68	1.00 (0.61 ~ 1.62)	5.4	-	-
Solomon et al. [137]	-	-	-	-	1.58 (1.22 ~ 2.06)	6.83	-	-
Mielke et al. [138]	-	-	-	-	2.82 (0.94 ~ 8.43)	0.14	-	-
Huang et al. [139]	-	-	-	-	1.06 (0.75 ~ 1.51)	7.65	-	-
Kivipelto et al. [140]	-	-	-	-	2.12 (1.05 ~ 4.30)	0.74	-	-
Reitz et al. [48]	-	-	-	-	0.80 (0.40 ~ 1.50)	4.79	-	-
Kimm et al. [141]	-	-	-	-	1.20 (1.00 ~ 1.50)	11.06	-	-
Kimm et al. [141]	-	-	-	-	1.10 (0.90 ~ 1.30)	12.6	-	-
Kivipelto et al. [142]	-	-	-	-	2.80 (1.20 ~ 6.70)	0.26	-	-
Hayden et al. [143]	-	-	-	-	0.47 (0.19 ~ 0.98)	7.33	-	-
Notkola et al. [144]	-	-	-	-	3.10 (1.20 ~ 8.50)	0.15	-	-
Ronnemaa et al. [145]	-	-	-	-	1.00 (0.90 ~ 1.20)	14.13	-	-
Wang et al. [146]	-	-	-	-	0.69 (0.66 ~ 1.32)	10.80	-	-
Lieb et al. [147]	-	-	1.60 (0.50 ~ 5.50)	0.3	0.80 (0.20 ~ 2.50)	1.41	-	-
Dal Forno et al. [148]	-	-	-	-	0.59 (0.23 ~ 1.53)	3.74	-	-
Dal Forno et al. [148]	-	-	-	-	0.35 (0.14 ~ 0.89)	7.76	-	-
Singh et al. [99]	-	-	-	-	1.15 (1.01 ~ 1.32)	15.70	-	-
Forti et al. [149]	-	-	0.56 (0.12 ~ 2.71)	1.14	-	-	-	-
Forti et al. [149]	-	-	0.83 (0.27 ~ 2.49)	1.55	-	-	-	-
Raffaitin et al. [150]	-	-	0.80 (0.27 ~ 2.49)	6.91	-	-	-	-
Muller et al. [151]	-	-	1.00 (0.70 ~ 1.40)	15.56	-	-	-	-
Singh et al. [99]	-	-	1.02 (0.86 ~ 1.19)	1	1.15 (1.01 ~ 1.32)	9.79	-	-
**Total effect sizes**	**68**		**53**		**81**		**34**	

References

1. Ban, Y.; Watanabe, T.; Suguro, T.; Matsuyama, T.; Iso, Y.; Sakai, T.; Sato, R.; Idei, T.; Nakano, Y.; Ota, H. Increased plasma urotensin-II and carotid atherosclerosis are associated with vascular dementia. *J. Atheroscler. Thromb.* **2009**, *16*, 179–187. https://doi.org/10.5551/jat.E608
2. Cacabelos, R.; Fernandez-Novoa, L.; Lombardi, V.; Corzo, L.; Pichel, V.; Kubota, Y. Cerebrovascular risk factors in Alzheimer's disease: brain hemodynamics and pharmacogenomic implications. *Neurol. Res.* **2003**, *25*, 567–580. https://doi.org/10.1179/016164103101202002
3. Caramelli, P.; Nitrini, R.; Maranhao, R.; Lourenço, A.C.G.; Damasceno, M.C.; Vinagre, C.; Caramelli, B. Increased apolipoprotein B serum concentration in Alzheimer's disease. *Acta Neurol. Scand.* **1999**, *100*, 61–63. https://doi.org/10.1111/j.1600-0404.1999.tb00724.x
4. Chen, H.; Du, Y.; Liu, S.; Ge, B.; Ji, Y.; Huang, G. Association between serum cholesterol levels and Alzheimer's disease in China: A case-control study. *Int. J. Food Sci. Nutr.* **2019**, *70*, 405–411. https://doi.org/10.1080/09637486.2018.1508426
5. Hoshino, T.; Kamino, K.; Matsumoto, M. Gene dose effect of the APOE-ϵ4 allele on plasma HDL cholesterol level in patients with Alzheimer's disease. *Neurobiol. Aging* **2002**, *23*, 41–45. https://doi.org/10.1016/S0197-4580(01)00252-4
6. Kouzuki, M.; Nagano, M.; Suzuki, T.; Katsumata, Y.; Nakamura, S.; Takamura, A.; Urakami, K. Cerebrospinal fluid biomarkers of Alzheimer's disease are associated with carotid plaque score and hemodynamics in intra-and extra-cranial arteries on ultrasonography. *J. Clin. Neurosci.* **2018**, *49*, 32–36. https://doi.org/10.1016/j.jocn.2017.12.006
7. Kuo, Y.-M.; Emmerling, M.R.; Bisgaier, C.L.; Essenburg, A.D.; Lampert, H.C.; Drumm, D.; Roher, A.E. Elevated low-density lipoprotein in Alzheimer's disease correlates with brain Aβ 1–42 levels. *Biochem. Biophys. Res. Commun.* **1998**, *252*, 711–715. https://doi.org/10.1006/bbrc.1998.9652
8. Lehtonen, A.; Luutonen, S. High-density lipoprotein cholesterol levels of very old people in the diagnosis of dementia. *Age Ageing* **1986**, *15*, 267–270. https://doi.org/10.1093/ageing/15.5.267
9. Lesser, G.; Kandiah, K.; Libow, L.S.; Likourezos, A.; Breuer, B.; Marin, D.; Mohs, R.; Haroutunian, V.; Neufeld, R. Elevated serum total and LDL cholesterol in very old patients with Alzheimer's disease. *Dement. Geriatr. Cogn. Disord.* **2001**, *12*, 138–145. https://doi.org/10.1159/000051248
10. Macesic, M.; Lalic, N.; Kostic, V.; Jotic, A.; Lalic, K.; Stefanova, E.; Milicic, T.; Lukic, L.; Stanarcic Gajovic, J.; Krako, N. Impaired insulin sensitivity and secretion in patients with Alzheimer's disease: the relationship with other atherosclerosis risk factors. *Curr. Vasc. Pharmacol.* **2017**, *15*, 158–166. https://doi.org/10.2174/1570161114666160905170644
11. Mamo, J.C.L.; Jian, L.; James, A.P.; Flicker, L.; Esselmann, H.; Wiltfang, J. Plasma lipoprotein β-amyloid in subjects with Alzheimer's disease or mild cognitive impairment. *Ann. Clin. Biochem.* **2008**, *45*, 395–403. https://doi.org/10.1258/acb.2008.007214
12. Panza, F.; Solfrizzi, V.; Colacicco, A.M.; Basile, A.M.; D'Introno, A.; Capurso, C.; Sabba, M.; Capurso, S.; Capurso, A. Apolipoprotein E (APOE) polymorphism influences serum APOE levels in Alzheimer's disease patients and centenarians. *Neuroreport* **2003**, *14*, 605–608. https://doi.org/10.1097/00001756-200303240-00016
13. Paragh, G.; Balla, P.; Katona, E.; Seres, I.; Égerházi, A.; Degrell, I. Serum paraoxonase activity changes in patients with Alzheimer's disease and vascular dementia. *Eur. Arch. Psychiatry Clin. Neurosci.* **2002**, *252*, 63–67. https://doi.org/10.1007/s004060200013
14. Ryglewicz, D.; Rodo, M.; Kunicki, P.K.; Bednarska-Makaruk, M.; Graban, A.; Lojkowska, W.; Wehr, H. Plasma antioxidant activity and vascular dementia. *J. Neurol. Sci.* **2002**, *203*, 195–197. https://doi.org/10.1016/S0022-510X(02)00290-3
15. Scacchi, R.; De Bernardini, L.; Mantuano, E.; Vilardo, T.; Donini, L.M.; Ruggeri, M.; Gemma, A.T.; Pascone, R.; Corbo, R.M. DNA polymorphisms of apolipoprotein B and angiotensin I-converting enzyme genes and

relationships with lipid levels in Italian patients with vascular dementia or Alzheimer's disease. *Dement. Geriatr. Cogn. Disord.* **1998**, *9*, 186–190. https://doi.org/10.1159/000017045

16. Shafagoj, Y.A.; Naffa, R.G.; El-Khateeb, M.S.; Abdulla, Y.L.; Al-Qaddoumi, A.A.; Khatib, F.A.; AL-Motassem, Y.F.; Al-Khateeb, E.M. APOE Gene polymorphism among Jordanian Alzheimer's patients with relation to lipid profile. *Neurosciences* **2018**, *23*, 29–34. https://doi.org/10.17712/nsj.2018.1.20170169
17. Solfrizzi, V.; Panza, F.; D'introno, A.; Colacicco, A.M.; Capurso, C.; Basile, A.M.; Capurso, A. Lipoprotein (a), apolipoprotein E genotype, and risk of Alzheimer's disease. *J. Neurol. Neurosurg. Psychiatry* **2002**, *72*, 732–736. https://doi.org/10.1136/jnnp.72.6.732
18. Tang, Y.; Li, Y.-M.; Zhang, M.; Chen, Y.-Q.; Sun, Q. ε3/4 genotype of the apolipoprotein E is associated with higher risk of Alzheimer's disease in patients with type 2 diabetes mellitus. *Gene* **2019**, *703*, 65–70. https://doi.org/10.1016/j.gene.2019.03.024
19. Warren, M.W.; Hynan, L.S.; Weiner, M.F. Lipids and adipokines as risk factors for Alzheimer's disease. *J. Alzheimers Dis.* **2012**, *29*, 151–157. https://doi.org/10.3233/JAD-2012-111385
20. Watanabe, T.; Miyazaki, A.; Katagiri, T.; Yamamoto, H.; Idei, T.; Iguchi, T. Relationship between serum insulin-like growth factor-1 levels and Alzheimer's disease and vascular dementia. *J. Am. Geriatr. Soc.* **2005**, *53*, 1748–1753. https://doi.org/10.1111/j.1532-5415.2005.53524.x
21. Wehr, H.; Bednarska-Makaruk, M.; Lojkowska, W.; Graban, A.; Hoffman-Zacharska, D.; Kuczyńska-Zardzewialy, A.; Mrugala, J.; Rodo, M.; Bochyńska, A.; Sulek, A. Differences in risk factors for dementia with neurodegenerative traits and for vascular dementia. *Dement. Geriatr. Cogn. Disord.* **2006**, *22*, 1–7. https://doi.org/10.1159/000092845
22. Wolf, H.; Hensel, A.; Arendt, T.; Kivipelto, M.; Winblad, B.; Gertz, H.-J. Serum lipids and hippocampal volume: the link to Alzheimer's disease? *Ann. Neurol. Off. J. Am. Neurol. Assoc. Child Neurol. Soc.* **2004**, *56*, 745–749. https://doi.org/10.1002/ana.20289
23. Yamamoto, H.; Watanabe, T.; Miyazaki, A.; Katagiri, T.; Idei, T.; Iguchi, T.; Mimura, M.; Kamijima, K. High Prevalence of Chlamydia Pneumoniae Antibodies and Increased High-Sensitive C-Reactive Protein in Patients with Vascular Dementia. *J. Am. Geriatr. Soc.* **2005**, *53*, 583–589. https://doi.org/10.1111/j.1532-5415.2005.53204.x
24. Yavuz, B.B.; Yavuz, B.; Halil, M.; Cankurtaran, M.; Ulger, Z.; Cankurtaran, E.S.; Aytemir, K.; Ariogul, S. Serum elevated gamma glutamyltransferase levels may be a marker for oxidative stress in Alzheimer's disease. *Int. Psychogeriatr.* **2008**, *20*, 815–823. https://doi.org/10.1017/S1041610208006790
25. Kalman, J.; Kudchodkar, B.J.; Murray, K.; McConathy, W.J.; Juhasz, A.; Janka, Z.; Lacko, A.G. Evaluation of serum-lipid-related cardiovascular risk factors in Alzheimer's disease. *Dement. Geriatr. Cogn. Disord.* **1999**, *10*, 488–493. https://doi.org/10.1159/000017195
26. Merched, A.; Xia, Y.; Visvikis, S.; Serot, J.M.; Siest, G. Decreased high-density lipoprotein cholesterol and serum apolipoprotein AI concentrations are highly correlated with the severity of Alzheimer's disease☆. *Neurobiol. Aging* **2000**, *21*, 27–30. https://doi.org/10.1016/S0197-4580(99)00103-7
27. Lesser, G.T.; Haroutunian, V.; Purohit, D.P.; Beeri, M.S.; Schmeidler, J.; Honkanen, L.; Neufeld, R.; Libow, L.S. Serum lipids are related to Alzheimer's pathology in nursing home residents. *Dement. Geriatr. Cogn. Disord.* **2009**, *27*, 42–49. https://doi.org/10.1159/000189268
28. Sun, X.; Shao, H.; Yu, D.; Wang, D. Analysis of correlation between insulin resistance, blood lipids and Alzheimer's disease. *Wei Sheng Yan Jiu/Journal of Hygiene Research* **2010**, *5*, 573–575.
29. Presečki, P.; Mück-Šeler, D.; Mimica, N.; Pivac, N.; Mustapić, M.; Stipčević, T.; Folnegović Šmalc, V. Serum lipid levels in patients with Alzheimer's disease. *Coll. Antropol.* **2011**, *35*, 115–120. https://doi:10.1016/S0197-4580(99)00103-7.
30. Parnowski, T.; Kaluza, B. Metabolic syndrome and cognitive dysfunction in the old age. *Psychiatr. Pol.* **2013**, *47*: 6, 1087–1099.
31. Grossi, M.F.; Carvalho, M. das G.; Silveira, J.N.; Gonçalves, G.S.; Gomes, K.B.; Bicalho, M.A.; Silva, I. de F.O. OxLDL plasma levels in patients with Alzheimer's disease. *Arq. Neuropsiquiatr.* **2018**, *76*, 241–246. https://doi.org/10.1590/0004-282x20180012
32. Agarwal, R.; Talwar, P.; Kushwaha, S.S.; Tripathi, C.B.; Kukreti, R. Effect of apolipoprotein E (APO E) polymorphism on leptin in Alzheimer's disease. *Ann. Indian Acad. Neurol.* **2015**, *18*, 320. https://doi.org/10.4103/0972-2327.157255

33. Alam, R.; Tripathi, M.; Mansoori, N.; Parveen, S.; Luthra, K.; Lakshmy, R.; Sharma, S.; Arulselvi, S.; Mukhopadhyay, A.K. Synergistic epistasis of paraoxonase 1 (rs662 and rs85460) and apolipoprotein E4 genes in pathogenesis of Alzheimer's disease and vascular dementia. *Am. J. Alzheimers Dis. Dementias®* **2014**, *29*, 769–776. https://doi.org/10.1177/1533317514539541
34. Cankurtaran, M.; Yavuz, B.B.; Halil, M.; Dagli, N.; Cankurtaran, E.S.; Ariogul, S. Are serum lipid and lipoprotein levels related to dementia? *Arch. Gerontol. Geriatr.* **2005**, *41*, 31–39. https://doi.org/10.1016/j.archger.2004.10.008
35. Chang, L.; Wang, Y.; Ji, H.; Dai, D.; Xu, X.; Jiang, D.; Hong, Q.; Ye, H.; Zhang, X.; Zhou, X. Elevation of peripheral BDNF promoter methylation links to the risk of Alzheimer's disease. *PloS One* **2014**, 9. https://doi.org/10.1371/journal.pone.0110773
36. Duan, D.; Ling, Y.; Zhou, Y. A clinical study on the serum triglyceride and total cholesterol of patients with vascular dementia and Alzheimer's disease. *Chin. J. Prev. Control Chronic Dis.* **2009**, *2*, 153-154.
37. Han, J.F.; Qu, Q.M.; Guo, F.; Yang, J.B.; Qiao, J.; Wu, C.B.; Ma, A.Q. Correlation between the levels of serum lipids and the onset of disease in patients with Alzheimer disease: 1:1 paired observation. *Chinese Journal of Clinical Rehabilitation* **2005**, *9,* 32-34.
38. Li, W. Relationship between changes of blood lipid metabolism and disease in patients with Alzheimer's disease. *Medical Information* **2014**, *27*.
39. Liu, J.H.; Chen, Z.L. Observation of serum cholesterol IL-6 and VitB12 levels in Alzheimer's disease or vascular dementia patients. *J. Jiangsu Univ. Med. Ed.* **2006**, *1*, 48–50.
40. Liu, Z.S. Study of lipid metabolism in patients with sporadic Alzheimer's disease. *China J Nerv Ment Dis* **2005**, *31*, 383-384.
41. Raygani, A.V.; Rahimi, Z.; Kharazi, H.; Tavilani, H.; Pourmotabbed, T. Association between apolipoprotein E polymorphism and serum lipid and apolipoprotein levels with Alzheimer's disease. *Neurosci. Lett.* **2006**, *408*, 68–72. https://doi.org/10.1016/j.neulet.2006.08.048
42. Shim, Y.S. Elevated remnant lipoprotein cholesterol in patients with Alzheimer's disease and vascular dementia: a pilot study. *Dementia and Neurocognitive Disorders,* **2010**, *7*.
43. Singh, N.K.; Chhillar, N.; Banerjee, B.D.; Bala, K.; Mukherjee, A.K.; Mustafa, M.D.; Mitrabasu Gene–environment interaction in Alzheimer's disease. *Am. J. Alzheimers Dis. Dementias®* **2012**, *27*, 496–503. https://doi.org/10.1177/1533317512456067
44. Sun, Y.L. Observation of blood lipid levels in patients with Alzheimer's disease. *China J Misdiagn* **2006**, *23*, 4542-4544.
45. Vasantharekha, R.; Priyanka, H.P.; Swarnalingam, T.; Srinivasan, A.V.; ThyagaRajan, S. Interrelationship between Mini-Mental State Examination scores and biochemical parameters in patients with mild cognitive impairment and Alzheimer's disease. *Geriatr. Gerontol. Int.* **2017**, *17*, 1737–1745. https://doi.org/10.1111/ggi.12957
46. Wada, H. Analyses of serum concentrations of apolipoproteins in the demented elderly. *Intern. Med.* **2000**, *39*, 220–222. https://doi.org/10.2169/internalmedicine.39.220
47. Wang, C.Y.; Fang, Y-y.; Chu, W-z,; Wu, Q.; Du, H.; Han, J.; Qian, C-y. A clinical study of Alzheimer's disease and vascular dementia in serum cholesterol, Vit B12, and folate acid. *Chinese Journal of Nervous and mental disease* **2005**, *31:*3, 188-191.
48. Wang, H. Study on the combining assay of total antioxidant status, high sensitive Creactive protein and serum lipids in the diagnosis of Alzheimer's disease. *Laboratory Medicine* **2006**, *21*.
49. Wang, R.; Chen, Z.; Fu, Y.; Wei, X.; Liao, J.; Liu, X.; He, B.; Xu, Y.; Zou, J.; Yang, X Plasma cystatin c and high-density lipoprotein are important biomarkers of Alzheimer's disease and vascular dementia: a cross-sectional study. *Frontiers in aging neuroscience* **2017**, 9. https://doi.org/10.3389/fnagi.2017.00026
50. Wang, X.H.; Zhang, N. Changes of serum levels of thyroid hormones and blood lipid in patients with varying degrees of Alzheimer's disease and related risk factors. *Journal of Clinical Medicine in Practice* **2016**, *5*, 32-35.
51. Watanabe, T.; Koba, S.; Kawamura, M.; Itokawa, M.; Idei, T.; Nakagawa, Y.; Iguchi, T.; Katagiri, T. Small dense low-density lipoprotein and carotid atherosclerosis in relation to vascular dementia. *Metabolism* **2004**, *53*, 476–482. https://doi.org/10.1016/j.metabol.2003.11.020
52. 108. Xiao, Z.; Wang, J.; Chen, W.; Wang, P.; Zeng, H.; Chen, W. Association studies of several cholesterol-related genes (ABCA1, CETP and LIPC) with serum lipids and risk of Alzheimer's disease. *Lipids Health Dis.* **2012**, *11*, 163. https://doi.org/10.1186/1476-511X-11-163

53. Xiao hong, W.; Shuyun, Z.; Xiao zhong, Y. Relationship between lipid disorders and carotid artery atherosclerosis. *Mod. J. Integr. Tradit. Chin. West. Med.* **2010**, *17*, 2096-2097
54. Yang, C.Z.; Tian, J.Z.; Zhong, J. Changes of blood lipid in mild cognitive impairment and Alzheimer's disease. *Chinese Journal of Gerontology* **2007**, *6*, 545-548.
55. Yu, Z.; Li, W.; Hou, D.; Zhou, L.; Deng, Y.; Tian, M.; Feng, X. Relationship between adiponectin gene polymorphisms and late-onset Alzheimer's disease. *PloS One* **2015**, *10*,4. https://doi.org/10.1371/journal.pone.0125186
56. Yuan, Y.G.; Ye, Q.; Chen, Y.; Li, H.L.; Lu, R.; Mei, G.; Chen, Z.; Li, Y.; Gu, X.; Liu, Y.; Zhang, S.; Wu, R. A study of serum lipid concentrations and apolipoprotein E genotype among patients with senile depression and alzhemier disease. *Chin. Gen. Pract.* **2006**, *9: 2*, 106–108.
57. Yue, Y.H.; Zhou, X.H.; Palida, A.B. Analysis of low density lipoprotein receptor-related protein gene 766C/T polymorphism and the levels of serum lipids in patients with Alzheimer's disease in Xinjiang Uygurs. *China J Psychiatry* **2009**, *3*, 129-133.
58. Zengi, O.; Karakas, A.; Ergun, U.; Senes, M.; Inan, L.; Yucel, D. Urinary 8-hydroxy-2′-deoxyguanosine level and plasma paraoxonase 1 activity with Alzheimer's disease. *Clin. Chem. Lab. Med.* **2012**, *50*, 529–534. https://doi.org/10.1515/cclm.2011.792
59. Zhao, Z.; Zhou, H.; Peng, Y.; Qiu, C.H.; Sun, Q.Y.; Wang, F.; Xie, H.N. Expression and significance of plasma 3-NT and ox-LDL in patients with Alzheimer's. *Genet. Mol. Res.* **2014**, *13*, 8428–8435. https://doi.org/10.4238/2014.October.20.19
60. Zheng, J.; Yan, H.; Shi, L.; Kong, Y.; Zhao, Y.; Xie, L.; Li, J.; Huang, M.; Li, J.; Zhao, S. The CYP19A1 rs3751592 variant confers susceptibility to Alzheimer disease in the Chinese Han population. *Medicine (Baltimore)* **2016**, 95. https://doi.org/10.1097/MD.0000000000004742
61. Zhong, X.; Yinglan, L.I.; Can, D.U.; Guofeng, L.I.; Hongjuan, L.I.; Zhu, A. Changes in serum homocysteine and its correlation with altitude, folacin and high-sensitivity C-reactive protein in Tibetan patients with mild-to-moderate Alzheimer's disease at different altitudes. *Chin. J. Geriatr.* **2016**, *35*, 934–938. https://doi.org/10.1515/CCLM.2011.792
62. Zhou, T. Comparative study on serum inflammatory cytokines and blood lipids in Alzheimer's disease and varcular dementia. *Medical Journal of Chinese People's Health* **2015**, *11*, 4-5.
63. Zhu, J.Y. Comparison of serum lipids, serum CRP, Hcy and sIL-6R in patients with Alzheimer's disease and those of the same age. *Pract Geriatr* **2007**, *21*.
64. Papassotiropoulos, A.; Lütjohann, D.; Bagli, M.; Locatelli, S.; Jessen, F.; Buschfort, R.; Ptok, U.; Björkhem, I.; Von Bergmann, K.; Heun, R. 24S-hydroxycholesterol in cerebrospinal fluid is elevated in early stages of dementia. *J. Psychiatr. Res.* **2002**, *36*, 27–32. https://doi.org/10.1016/S0022-3956(01)00050-4
65. Martínez-Morillo, E.; Hansson, O.; Atagi, Y.; Bu, G.; Minthon, L.; Diamandis, E.P.; Nielsen, H.M. Total apolipoprotein E levels and specific isoform composition in cerebrospinal fluid and plasma from Alzheimer's disease patients and controls. *Acta Neuropathol. (Berl.)* **2014**, *127*, 633–643. https://doi.org/10.1007/s00401-014-1266-2
66. Kölsch, H.; Lütjohann, D.; Jessen, F.; Urbach, H.; Von Bergmann, K.; Maier, W.; Heun, R. Polymorphism in neuropeptide Y influences CSF cholesterol levels but is no major risk factor of Alzheimer's disease. *J. Neural Transm.* **2006**, *113*, 231–238. https://doi.org/10.1007/s00702-005-0319-z
67. Kölsch, H.; Lütjohann, D.; Jessen, F.; Popp, J.; Hentschel, F.; Kelemen, P.; Schmitz, S.; Maier, W.; Heun, R. CYP46A1 variants influence Alzheimer's disease risk and brain cholesterol metabolism. *Eur. Psychiatry* **2009**, *24*, 183–190. https://doi.org/10.1016/j.eurpsy.2008.12.005
68. Kölsch, H.; Lütjohann, D.; Jessen, F.; Popp, J.; Hentschel, F.; Kelemen, P.; Friedrichs, S.; Maier, T.A.W.; Heun, R. RXRA gene variations influence Alzheimer's disease risk and cholesterol metabolism. *J. Cell. Mol. Med.* **2009**, *13*, 589–598. https://doi.org/10.1111/j.1582-4934.2009.00383.x
69. Kölsch, H.; Heun, R.; Jessen, F.; Popp, J.; Hentschel, F.; Maier, W.; Lütjohann, D. Alterations of cholesterol precursor levels in Alzheimer's disease. *Biochim. Biophys. Acta BBA - Mol. Cell Biol. Lipids* **2010**, *1801*, 945–950. https://doi.org/10.1016/j.bbalip.2010.03.001
70. Qureischie, H.; Heun, R.; Lütjohann, D.; Popp, J.; Jessen, F.; Ledschbor-Frahnert, C.; Thiele, H.; Maier, W.; Hentschel, F.; Kelemen, P.; et al. CETP polymorphisms influence cholesterol metabolism but not Alzheimer's disease risk. *Brain Res.* **2008**, *1232*, 1–6. https://doi.org/10.1016/j.brainres.2008.07.047

71. Mateos, L.; Ismail, M.-A.-M.; Gil-Bea, F.-J.; Leoni, V.; Winblad, B.; Björkhem, I.; Cedazo-Mínguez, A. Upregulation of Brain Renin Angiotensin System by 27-Hydroxycholesterol in Alzheimer's Disease. *J. Alzheimers Dis.* **2011**, *24*, 669–679. https://doi.org/10.3233/JAD-2011-101512
72. Wollmer, M.A.; Streffer, J.R.; Tsolaki, M.; Grimaldi, L.M.E.; Lütjohann, D.; Thal, D.; von Bergmann, K.; Nitsch, R.M.; Hock, C.; Papassotiropoulos, A. Genetic association of acyl-coenzyme A: cholesterol acyltransferase with cerebrospinal fluid cholesterol levels, brain amyloid load, and risk for Alzheimer's disease. *Mol. Psychiatry* **2003**, *8*, 635–638. https://doi.org/10.1038/sj.mp.4001296
73. Wollmer, M.A.; Streffer, J.R.; Lütjohann, D.; Tsolaki, M.; Iakovidou, V.; Hegi, T.; Pasch, T.; Jung, H.H.; Bergmann, K. von; Nitsch, R.M.; et al. ABCA1 modulates CSF cholesterol levels and influences the age at onset of Alzheimer's disease. *Neurobiol. Aging* **2003**, *24*, 421–426. https://doi.org/10.1016/S0197-4580(02)00094-5
74. Shafaati, M.; Solomon, A.; Kivipelto, M.; Björkhem, I.; Leoni, V. Levels of ApoE in cerebrospinal fluid are correlated with Tau and 24S-hydroxycholesterol in patients with cognitive disorders. *Neurosci. Lett.* **2007**, *425*, 78–82. https://doi.org/10.1016/j.neulet.2007.08.014
75. Schönknecht, P.; Lütjohann, D.; Pantel, J.; Bardenheuer, H.; Hartmann, T.; von Bergmann, K.; Beyreuther, K.; Schröder, J. Cerebrospinal fluid 24S-hydroxycholesterol is increased in patients with Alzheimer's disease compared to healthy controls. *Neurosci. Lett.* **2002**, *324*, 83–85. https://doi.org/10.1016/S0304-3940(02)00164-7
76. Popp, J.; Lewczuk, P.; Kölsch, H.; Meichsner, S.; Maier, W.; Kornhuber, J.; Jessen, F.; Lütjohann, D. Cholesterol metabolism is associated with soluble amyloid precursor protein production in Alzheimer's disease. *J. Neurochem.* **2012**, *123*, 310–316. https://doi.org/10.1111/j.1471-4159.2012.07893.x
77. Popp, J.; Meichsner, S.; Kölsch, H.; Lewczuk, P.; Maier, W.; Kornhuber, J.; Jessen, F.; Lütjohann, D. Cerebral and extracerebral cholesterol metabolism and CSF markers of Alzheimer's disease. *Biochem. Pharmacol.* **2013**. https://doi:10.1016/j.neulet.2007.08.014.
78. Vanmierlo, T.; Popp, J.; Kölsch, H.; Friedrichs, S.; Jessen, F.; Stoffel-Wagner, B.; Bertsch, T.; Hartmann, T.; Maier, W.; Bergmann, K. von; et al. The plant sterol brassicasterol as additional CSF biomarker in Alzheimer's disease. *Acta Psychiatr. Scand.* **2011**, *124*, 184–192. https://doi.org/10.1111/j.1600-0447.2011.01713.x
79. Leoni, V.; Caccia, C. Potential diagnostic applications of side chain oxysterols analysis in plasma and cerebrospinal fluid. *Biochem. Pharmacol.* **2013**, *86*, 26–36. https://doi.org/10.1016/j.bcp.2013.03.015
80. Li, G.; Shofer, J.B.; Kukull, W.A.; Peskind, E.R.; Tsuang, D.W.; Breitner, J.C.; McCormick, W.; Bowen, J.D.; Teri, L.; Schellenberg, G.D. Serum cholesterol and risk of Alzheimer disease: a community-based cohort study. *Neurology* **2005**, *65*, 1045–1050. https://doi.org/10.1212/01.wnl.0000178989.87072.11
81. Solomon, A.; Kivipelto, M.; Wolozin, B.; Zhou, J.; Whitmer, R.A. Midlife serum cholesterol and increased risk of Alzheimer's and vascular dementia three decades later. *Dement. Geriatr. Cogn. Disord.* **2009**, *28*, 75–80. https://doi.org/10.1159/000231980
82. Mielke, M.M.; Zandi, P.P.; Shao, H.; Waern, M.; Östling, S.; Guo, X.; Björkelund, C.; Lissner, L.; Skoog, I.; Gustafson, D.R. The 32-year relationship between cholesterol and dementia from midlife to late life. *Neurology* **2010**, *75*, 1888–1895. https://doi.org/10.1212/WNL.0b013e3181feb2bf
83. Huang, C.-C.; Chung, C.-M.; Leu, H.-B.; Lin, L.-Y.; Chiu, C.-C.; Hsu, C.-Y.; Chiang, C.-H.; Huang, P.-H.; Chen, T.-J.; Lin, S.-J. Diabetes mellitus and the risk of Alzheimer's disease: a nationwide population-based study. *PloS One* **2014**, *9*. https://doi.org/10.1371/journal.pone.0087095
84. Kivipelto, M.; Ngandu, T.; Fratiglioni, L.; Viitanen, M.; K\aareholt, I.; Winblad, B.; Helkala, E.-L.; Tuomilehto, J.; Soininen, H.; Nissinen, A. Obesity and vascular risk factors at midlife and the risk of dementia and Alzheimer disease. *Arch. Neurol.* **2005**, *62*, 1556–1560. https://doi.org/10.1001/archneur.62.10.1556
85. Kimm, H.; Lee, P.H.; Shin, Y.J.; Park, K.S.; Jo, J.; Lee, Y.; Kang, H.C.; Jee, S.H. Mid-life and late-life vascular risk factors and dementia in Korean men and women. *Arch. Gerontol. Geriatr.* **2011**, *52*, e117–e122. https://doi.org/10.1016/j.archger.2010.09.004
86. Kivipelto, M.; Helkala, E.-L.; Laakso, M.P.; Hänninen, T.; Hallikainen, M.; Alhainen, K.; Iivonen, S.; Mannermaa, A.; Tuomilehto, J.; Nissinen, A. Apolipoprotein E ϵ4 allele, elevated midlife total cholesterol level, and high midlife systolic blood pressure are independent risk factors for late-life Alzheimer disease. *Ann. Intern. Med.* **2002**, *137*, 149–155. https://doi.org/10.7326/0003-4819-137-3-200208060-00006

87. Hayden, K.M.; Zandi, P.P.; Lyketsos, C.G.; Khachaturian, A.S.; Bastian, L.A.; Charoonruk, G.; Tschanz, J.T.; Norton, M.C.; Pieper, C.F.; Munger, R.G. Vascular risk factors for incident Alzheimer disease and vascular dementia: the Cache County study. *Alzheimer Dis. Assoc. Disord.* **2006**, *20*, 93–100. https://doi.org/10.1097/01.wad.0000213814.43047.86
88. Notkola IL,; Sulkava R,; Pekkanen J,; Erkinjuntti T,; Ehnholm C,; Kivinen P,; Tuomilehto J,; Nissinen. A Serum total cholesterol, apolipoprotein E epsilon 4 allele, and Alzheimer's disease. *Neuroepidemiology* **1998**, *17*, 14-20. https://doi.org/10.1159/000026149
89. Rönnemaa, E.; Zethelius, B.; Lannfelt, L.; Kilander, L. Vascular risk factors and dementia: 40-year follow-up of a population-based cohort. *Dement. Geriatr. Cogn. Disord.* **2011**, *31*, 460–466. https://doi.org/10.1159/000330020
90. Wang, K.-C.; Woung, L.-C.; Tsai, M.-T.; Liu, C.-C.; Su, Y.-H.; Li, C.-Y. Risk of Alzheimer's disease in relation to diabetes: a population-based cohort study. *Neuroepidemiology* **2012**, *38*, 237–244. https://doi.org/10.1159/000337428
91. Lieb W; Beiser AS; Vasan RS; Tan ZS; Au R; Harris TB; Roubenoff R; Auerbach S; DeCarli C; Wolf PA; et al. Association of plasma leptin levels with incident Alzheimer disease and MRI measures of brain aging. *JAMA* **2009**, *302*, 2565-2572. https://doi.org/10.1001/jama.2009.1836
92. Dal Forno, G.; Palermo, M.T.; Donohue, J.E.; Karagiozis, H.; Zonderman, A.B.; Kawas, C.H. Depressive symptoms, sex, and risk for Alzheimer's disease. *Ann. Neurol.* **2005**, *57*, 381–387. https://doi.org/10.1002/ana.20405
93. Forti, P.; Pisacane, N.; Rietti, E.; Lucicesare, A.; Olivelli, V.; Mariani, E.; Mecocci, P.; Ravaglia, G. Metabolic syndrome and risk of dementia in older adults. *J. Am. Geriatr. Soc.* **2010**, *58*, 487–492. https://doi.org/10.1111/j.1532-5415.2010.02731.x
94. Raffaitin, C.; Gin, H.; Empana, J.-P.; Helmer, C.; Berr, C.; Tzourio, C.; Portet, F.; Dartigues, J.-F.; Alpérovitch, A.; Barberger-Gateau, P. Metabolic syndrome and risk for incident Alzheimer's disease or vascular dementia: the Three-City Study. *Diabetes Care* **2009**, *32*, 169–174. https://doi.org/10.2337/dc08-0272
95. Muller, M.; Tang, M.-X.; Schupf, N.; Manly, J.J.; Mayeux, R.; Luchsinger, J.A. Metabolic syndrome and dementia risk in a multiethnic elderly cohort. *Dement. Geriatr. Cogn. Disord.* **2007**, *24*, 185–192. https://doi.org/10.1159/000105927
